# Investigating the Protective Effects of a Citrus Flavonoid on the Retardation Morphogenesis of the Oligodendroglia-like Cell Line by Rnd2 Knockdown

**DOI:** 10.3390/neurolint16010003

**Published:** 2023-12-26

**Authors:** Shoya Fukatsu, Yuki Miyamoto, Yu Oka, Maki Ishibashi, Remina Shirai, Yuki Ishida, Shin Endo, Hironori Katoh, Junji Yamauchi

**Affiliations:** 1 Laboratory of Molecular Neurology, Tokyo University of Pharmacy and Life Sciences, Tokyo 192-0392, Japan; s189072@toyaku.ac.jp (S.F.); miyamoto-y@ncchd.go.jp (Y.M.); rshirai@toyaku.ac.jp (R.S.); 2 Laboratory of Molecular Pharmacology, National Research Institute for Child Health and Development, Setagaya-ku, Tokyo 157-8535, Japan; 3 Personal Health Care Division, Hayashibara Co., Ltd., Okayama 702-8006, Japan; 4 Department of Biological Chemistry, Graduate School of Science, Osaka Metropolitan University, Osaka 599-8531, Japan; hirokato@omu.ac.jp; 5 Diabetic Neuropathy Project, Tokyo Metropolitan Institute of Medical Science, Setagaya-ku, Tokyo 156-8506, Japan

**Keywords:** monomeric GTP-binding proteins, phosphotransferases, signal transduction, disease, flavonoid, RNA, small interfering, clustered regularly interspaced short palindromic repeats, cell line, morphogenesis, oligodendroglia

## Abstract

Recent discoveries suggest links between abnormalities in cell morphogenesis in the brain and the functional deficiency of molecules controlling signal transduction in glial cells such as oligodendroglia. Rnd2 is one such molecule and one of the Rho family monomeric GTP-binding proteins. Despite the currently known functions of Rnd2, its precise roles as it relates to cell morphogenesis and disease state remain to be elucidated. First, we showed that signaling through the loss of function of the *rnd2* gene affected the regulation of oligodendroglial cell-like morphological differentiation using the FBD-102b cell line, which is often utilized as a differentiation model. The knockdown of Rnd2 using the clustered regularly interspaced palindromic repeats (CRISPR)/CasRx system or RNA interference was shown to slow morphological differentiation. Second, the knockdown of Prag1 or Fyn kinase, a signaling molecule acting downstream of Rnd2, slowed differentiation. Rnd2 or Prag1 knockdown also decreased Fyn phosphorylation, which is critical for its activation and for oligodendroglial cell differentiation and myelination. Of note, hesperetin, a citrus flavonoid with protective effects on oligodendroglial cells and neurons, can recover differentiation states induced by the knockdown of Rnd2/Prag1/Fyn. Here, we showed that signaling through Rnd2/Prag1/Fyn is involved in the regulation of oligodendroglial cell-like morphological differentiation. The effects of knocking down the signaling cascade molecule can be recovered by hesperetin, highlighting an important molecular structure involved in morphological differentiation.

## 1. Introduction

Oligodendrocytes (also called oligodendroglia or oligodendroglial cells) are central nervous system (CNS) glial cells that play a critical role in forming the myelin sheath [1,2,3,4]. Recent studies on function and dysfunction in the CNS show that various intracellular and extracellular signaling molecules and molecules modulating cell morphogenesis in oligodendroglial cells are associated with hypomyelinating and/or demyelinating diseases, inflammatory diseases, and neuropsychiatric disorders [1,2,3,4]. For example, the molecular pathways that are dysregulated in neuropsychiatric disorders include glutamate homeostasis, cell–cell interaction through various metabolites, and inflammation, all of which are also regulated by glial cells and their morphogenesis [1,2].

The molecules underlying neuropsychiatric disorders include small GTPases, some of which mediate cell morphological changes. Rnd2 is one such small GTPase, belonging to an atypical member of the Rho family small GTPase, and loss of function or functional inhibition of Rnd2 may be related to their disorders [5,6,7,8]. Similar to Ras GTPases, Rho GTPases are generally bound to GTP or GDP. They normally function as on and off switches for signal transducers to control various situations in many cell types [9,10,11,12]. The GTP-binding form is active in Rho GTPases and is generated by the guanine nucleotide exchange factor (GEF), replacing intracellular GDP with GTP [9,10,11,12]. The GDP-binding form is inactive and is catalyzed by the GTPase-activating protein (GAP) [9,10,11,12]. However, because the Rnd family members Rnd1, Rnd2, and Rnd3 have little or no GTPase activity, guanine nucleotide switching reactions are unlikely to occur. Therefore, cellular activities of the Rnd family members, including those of Rnd2, are thought to be regulated at the transcriptional or posttranslational levels; that is, expression levels may control Rnd family activities [13,14].

The Rnd family members are known to bind and activate unique effector proteins such as GAP for RhoA (a typical Rho GTPase member), resulting in the promotion of cell morphogenesis through the inhibition of RhoA [13,14,15,16]. In addition, Rnd1 and Rnd3 are ubiquitously expressed throughout tissues and cell types, whereas Rnd2 appears to have limited expression, especially in brain tissues [13,14]. This may also be related to the fact that Rnd2 has different effectors from Rnd1 and Rnd3. To date, Prag1 appears to be a unique effector for Rnd2. Prag1 acts as an adaptor protein stimulating the enzymatic activity of Fyn non-receptor-type tyrosine kinase by insulating Csk, which is the negative regulator of Fyn tyrosine kinase [13,14,15,16].

Before wrapping neuronal axons with myelin sheaths, oligodendroglial precursor cells differentiate into mature oligodendroglial cells. It is well known that the molecular mechanisms underlying the differentiation into mature oligodendroglial cells require many specific identified nuclear transcription factors [17,18,19,20]. The Sox family transcription factors Sox9 and Sox10 and the Olig family transcription factors Olig1 and Olig2 are expressed at almost all oligodendroglial cell developmental stages, including the initial stages of the oligodendroglial precursor cell [17,18,19,20]. These transcription factors participate not only in determining the differentiating direction of oligodendroglial precursor cells but also in becoming mature oligodendroglial cells and, in turn, myelinating ones [17,18,19,20]. Even though the Sox and Olig family transcription factors are expressed at oligodendroglial cell developmental stages, there are transcription factors that specifically control the fate of more mature cells. For example, myelin regulatory factor (MRF) is required for stages such as oligodendroglial cell maturation, differentiation, and myelination [17,18,19,20].

The transcription factors, including the Sox and Olig family transcription factors and MRF, cooperatively contribute to the expression of differentiation and myelination marker proteins [17,18,19]. The representative marker proteins are the proteolipid protein 1 (PLP1) and 2′, 3′-cyclic nucleotide 3′-phosphodiesterase (CNPase) [17,18,19]. It is known that precursor cells differentiate into further lineage cells by an array of transcription factor molecules called timers, which are centered around a network of transcription factors inside cells; however, intracellular molecules may constitute the signaling molecular network that resides in the transcription factor niche [17,18,19,20]. The limited information on intracellular signaling molecules describes the roles of Fyn tyrosine kinase and Akt serine/threonine kinase in maintaining activities and/or expression levels of transcription factors during myelination [17,18,19]. Abnormal regulation of or defects in such molecules is closely linked to oligodendroglial cell- and myelin-related disorders and some neurodevelopmental diseases [21,22,23,24].

Using immunohistochemical and immunoblotting techniques, we previously demonstrated that Rnd2 is primarily expressed in oligodendroglial cells in the brain [25]. This finding suggested the role of oligodendroglial cells. Furthermore, the *rnd2* gene, encoding Rnd2, was one of the genes upregulated in primary oligodendroglial cells following the induction of differentiation. Despite the unique regulation of Rnd2 in oligodendroglial cell myelination [25], the question of whether Rnd2 itself has the ability to trigger differentiation in oligodendroglial cells before wrapping axons with their differentiated plasma membranes remains to be elucidated. Thus, we explored whether signaling through Rnd2 is involved in regulating the formation of differentiation phenotypes with widespread plasma membranes using the FBD-102b cell line. This cell line is a commonly used mouse oligodendroglial cell-like differentiation model, derived from a p53 knockout mouse-isolated single glial cell [26,27]. In addition to the FBD-102b cells, CG4 cells are characterized as a rat precursor cell line that has the ability to moderately differentiate [28,29]. Because FBD-102b cells are derived from mice, they can also be used to emphasize the reciprocity of the results with genetically modified mice [25]. In contrast, rodent primary oligodendroglial cell precursor cells display differentiation phenotypes with dynamically widespread plasma membranes, although it takes a lot of time and a variety of transfection studies to establish such primary precursor cells with high purity [17,18,19,20,25]. Therefore, using the FBD-102b cell system, we examined whether Rnd2 and the related signaling molecules were involved in promoting morphological differentiation. We also explored whether hesperetin, a major citrus flavonoid in dried citrus peel (*chinpi*) that has protective effects on oligodendroglial and neuronal cells [30], could have the ability to affect differentiation states induced by the knockdown of the respective molecules constituting signaling through Rnd2.

## 2. Materials and Methods

### 2.1. Study Design

The purpose of this study is to examine whether Rnd2 and the downstream signaling molecules Prag1 and Fyn are involved in the regulation of oligodendroglial cell morphological differentiation. Therefore, we transfected FBD-102b cells with siRNA and/or Grna of target molecules and assayed both cell morphological changes and expression levels of differentiation markers. We also measured Fyn phosphorylation levels downstream of Rnd2 using antibodies specific for the phosphorylation states. We further tested whether hesperetin has protective effects on cell morphogenesis induced by knockdown of Rnd2 or the downstream signaling molecules Prag1 or Fyn.

### 2.2. Antibodies and Other Materials

The key materials such as antibodies and chemicals used in this experiment are listed in Table 1.

### 2.3. Synthetic Small Interfering (si)RNAs, Guide (g)RNA-Encoding DNA Sequences, and DNA Primers

The 19-mer siRNAs with tandem deoxythymidine dinucleotides (dTdT) were synthesized by Fasmac (Kanagawa, Japan). The following sense and antisense siRNAs (with nucleotide numbers from A^1^UG in parentheses) were annealed in accordance with the manufacturer’s instructions [25,31]: Rnd2 (156) siRNA sense: 5′-GCGCCGCAUUGAGCUCAAC-dTdT-3′ and antisense: 5′-GUUGAGCUCAAUGCGGCGC-dTdT-3′; Rnd2 (300) siRNA sense: 5′-GUGGCAAGGAGAGACUCAG-dTdT-3′ and antisense: 5′-CUGAGUCUCUCCUUGCCAC-dTdT-3′; Prag1 (105) siRNA sense: 5′-GGCUAGAGCCAACAGCCUA-dTdT-3′ and antisense: 5′-UAGGCUGUUGGCUCUAGCC-dTdT-3′; Prag1 (178) siRNA sense: 5′-GGCGUGAAUGGCUUGGCCU-dTdT-3′ and antisense: 5′-AGGCCAAGCCAUUCACGCC-dTdT-3′; and Prag1 (204) siRNA sense: 5′-GCCCACCAUUGCUGUAAAG-dTdT-3′ and antisense: 5′-CUUUACAGCAAUGGUGGGC-dTdT-3′. Mouse Fyn siRNA was purchased from Santa Cruz Biotechnology (Cat. No. sc-35425; Santa Cruz, CA, USA). Control (*Photinus pyralis* luciferase) siRNA was described previously [31].

The following DNA sequences encoding sense and antisense gRNAs were annealed and inserted into pSINmU6 (Takara Bio Gene No. X06980) in accordance with the manufacturer’s instructions (nucleotide numbers from A^1^TG in parentheses) [25,31]: Rnd2 (113) Grna sense: 5′-GATCCGCACCCGTGCAAAAATGCAGGGGTCTAAAACCCACGGTGTTTGAGAACTACACTTTTTTAT-3′ and antisense: 5′-CGATAAAAAAGTGTAGTTCTCAAACACCGTGGGTTTTAGACCCCTGCATTTTTGCACGGGTGCG-3′; and Rnd2 (250) Grna sense: 5′-GATCCGCACCCGTGCAAAAATGCAGGGGTCTAAAACATCTGCTTTGACATTAGCCGGCTTTTTTAT-3′ and antisense: 5′-CGATAAAAAAGCCGGCTAATGTCAAAGCAGATGTTTTAGACCCCTGCATTTTTGCACGGGTGCG-3′. Control (*P. pyralis* luciferase) Grna was described previously [31].

The following DNA primer pairs, Rnd2 sense primer (melting temperature [Tm] values = 55 °C): 5′-ATGTGGGATACTTCAGGTTCC-3′ and antisense (Tm values = 56 °C): 5′-TCACATGAGGTTACAGCTCTTG-3′; Prag1 sense primer (Tm values = 62 °C): 5′-ATGTCTGCGTGCAGCGACTTTG-3′ and antisense (Tm values = 62 °C): 5′-CGGTTCCTTCACAAGAGAGCAGTAGTC-3′; and Fyn sense primer (Tm values = 61 °C): 5′-ATGGGCTGTGTGCAATGTAAGGATAAAG-3′ and antisense (Tm values = 60 °C): 5′-GGTTTGGCAACTTCAGAGCTCTTC-3′ were used in polymerase chain reaction (PCR) [25,26,27]. The control actin primer pair was described previously [31].

### 2.4. Reverse Transcription (RT)-Polymerase Chain Reaction (PCR)

The cDNAs [25,26,27] were prepared from Isogen (Nippon Gene, Tokyo, Japan)-extracted total RNA with the PrimeScript RT Master Mix kit (Takara Bio, Kyoto, Japan) in accordance with the manufacturer’s instructions.

PCR amplification from RT products [25] was performed using Gflex DNA polymerase (Takara Bio) with 30 to 36 cycles, each consisting of a denaturation reaction at 98 °C (0.2 min), an annealing reaction at 54.5 to 61.5 °C (0.25 min) depending on the annealing temperature, and an extension reaction at 68 °C (0.5 min). The resultant PCR products were loaded onto 1% to 2% agarose gels (Nacalai Tesque, Kyoto, Japan; Fujifilm, Tokyo, Japan).

### 2.5. Cell Cultures

FBD-102b cells, which belong to the mouse oligodendroglial precursor cell line [26,27], were cultured on cell culture dishes (Nunc brand, Thermo Fisher Scientific, Waltham, MA, USA) in Dulbecco’s modified Eagle’s medium (DMEM)/F-12 mixed medium (Nacalai Tesque) containing 10% heat-inactivated fetal bovine serum (FBS; Gibco brand, Thermo Fisher Scientific) and penicillin–streptomycin solution (Thermo Fisher Scientific) in 5% CO_2_ at 37 °C [26,27].

To induce differentiation, cells were cultured on polylysine (Nacalai Tesque)-coated cell culture dishes in culture medium without FBS for up to several days in 5% CO_2_ at 37 °C [26,27,31] in the presence or absence (vehicle control composed of only dimethyl sulfoxide [DMSO]) of hesperetin chemicals (15 Mm for hesperetin and 25 Mm for monoglucosyl hesperidine; Fujifilm). Cells with myelin membrane-like widespread membranes (cells large enough to contain a circle with a diameter of ≥50 mm) were considered to represent the differentiated phenotype. Following the induction of differentiation, expression levels of some marker proteins gradually increase, accompanied by widespread membrane formation [26,27,31]. Cell morphologies were captured using microscopic systems equipped with i-NTER LENS (Micronet, Saitama, Japan) and i-NTER software (Micronet). The sets of resultant images were analyzed with Image J software (ver. 2.15.0; URL: https://imagej.nih.gov/, accessed on 1 April 2022).

### 2.6. siRNA and Grna Transfection Techniques

Cells were transfected with the respective synthesized 21-mer siRNAs with dTdT or plasmids (Addgene Gene No. 237577 plasmid expressing CasRx and pSINmU6 encoding Grna) using the ScreenFect siRNA or ScreenFect A transfection kit (Fujifilm) in accordance with the manufacturer’s instructions, respectively [16,31]. The medium was replaced 4 h after transfection and generally used for ≥48 h after transfection for biochemical experiments. In these conditions, attached cells incorporating trypan blue (Nacalai Tesque) were estimated to be <5% in each experiment 48 h after transfection [16,31].

### 2.7. Polyacrylamide Gel Electrophoresis and Immunoblotting Following Immunoprecipitation

Cells were lysed in lysis buffer (50 Mm HEPES-NaOH, Ph 7.5, 150 Mm NaCl, 3 Mm MgCl_2_, 1 Mm dithiothreitol, 1 Mm phenylmethane sulfonylfluoride, 1 μg/ML leupeptin, 1 Mm EDTA, 1 Mm Na_3_VO_4_, 10 Mm NaF, and 0.5% NP-40; Nacalai Tesque) [15,16,31,32]. For denatured conditions, cell lysates were denatured in sample buffers (Fujifilm). The denatured samples and denatured immunoprecipitated complexes composed of primary antibody-captured antigen and Protein G resin (Thermo Fisher Scientific) were separated on premade sodium dodecylsulfate-polyacrylamide gel (Nacalai Tesque). The electrophoretically separated proteins were transferred to a polyvinylidene fluoride membrane (Fujifilm), blocked with Blocking One (Nacalai Tesque), and immunoblotted using primary antibodies, followed by peroxidase enzyme-conjugated secondary antibodies [15,16,31,32]. The peroxidase-reactive bands were exposed on X-ray films (Fujifilm), captured using an image scanner (Canon, Tokyo, Japan), and scanned using CanoScan software (Canon). We also used a chemiluminescence scanner (C-DiGit, LI-COR, Lincoln, NE, USA) and captured immunoreactive bands through Image Studio software (LI-COR). We performed some sets of experiments in immunoblotting studies and quantified other immunoreactive bands with Image J software ver. 2.15.0.

### 2.8. Statistical Analyses

Values are means ± SD from separate experiments. Intergroup comparisons were performed using the unpaired *t*-test with Student’s or Welch’s correction in Excel software (ver. 2019; Microsoft Corp., Redmond, WA, USA) [25,31]. Differences were considered significant at *p* < 0.05. For all analyses, the investigator was blinded to the sample conditions [25,31].

### 2.9. Ethics Statements

Techniques using genetically modified cells and related techniques were performed in accordance with a protocol approved by the Tokyo University of Pharmacy and Life Sciences Gene and Animal Care Committee (Approval Nos. LS28-20 and LSR3-011).

## 3. Results

### 3.1. Rnd2 Positively Regulates Oligodendroglial Cell Morphological Differentiation

**Figure 1:**
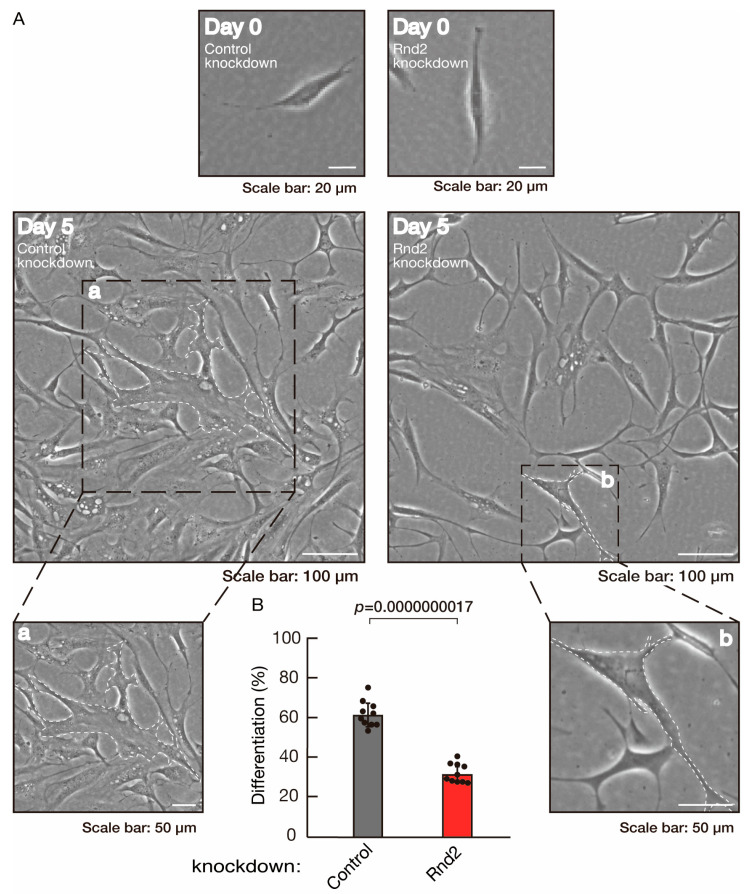
Knockdown of Rnd2 using the CRISPR/CasRx system slows morphological differentiation. (**A**,**B**) FBD-102b cells were transfected with the plasmids encoding CasRx and gRNA for control (luciferase) or Rnd2 for knocking down Rnd2. Following the induction of differentiation, the respective cell morphologies at 0 and 5 days were captured as the representative images, analyzed, and statistically depicted (*p* < 0.01; n = 10 fields). Cells with myelin membrane-like widespread membranes (cells large enough to contain a circle with a diameter of 50 mm or more) were considered to be differentiated. Typical cells with widespread membranes are surrounded by dashed lines. The inset images (a,b) are magnified from the squares in the large images.

**Figure 2:**
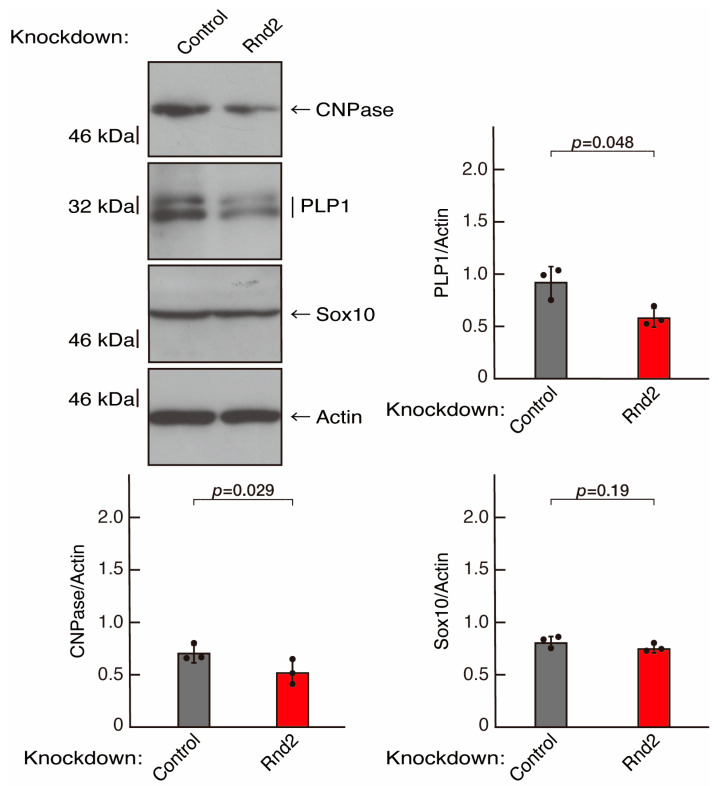
Knockdown of Rnd2 using the CRISPR/CasRx system decreases the expression levels of myelination/differentiation markers. FBD-102b cells were transfected with the plasmids encoding CasRx and gRNA for control (luciferase) or Rnd2. Following the induction of differentiation, the respective immunoblots (CNPase, PLP1, Sox10, and actin) at 5 days were captured as the representative images, analyzed, and statistically depicted as immunoreactive bands normalized to actin ones. (n = 3 blots).

To investigate whether Rnd2 is involved in the regulation of morphological differentiation, we knocked down Rnd2 using a CRISPR/CasRx system [33,34] in the mouse oligodendroglial FBD-102b cell line (Figure S1). We co-transfected plasmids encoding Grna suitable for the CRISPR/CasRx system and CasRx into FBD-102b cells. The knockdown of Rnd2 in cells resulted in the slowing of morphological differentiation, whereas the control (luciferase) knockdown achieved differentiation with oligodendroglial cell-like widespread membranes (Figure 1A,B). FBD-102b cells showed increased expression of some marker proteins following the induction of differentiation [26,27]. Then, we compared expression levels of oligodendroglial differentiation and myelination markers in Rnd2 and control knocked-down cells. Following the induction of differentiation, expression of the markers PLP1 and CNPase was decreased in Rnd2 knockdown cells, whereas expression of oligodendrocyte lineage marker Sox10 and control actin was comparable in control knockdown and Rnd2 knockdown cells (Figure 2), suggesting that Rnd2 positively mediates oligodendroglial cell-like morphological differentiation.

**Figure 3:**
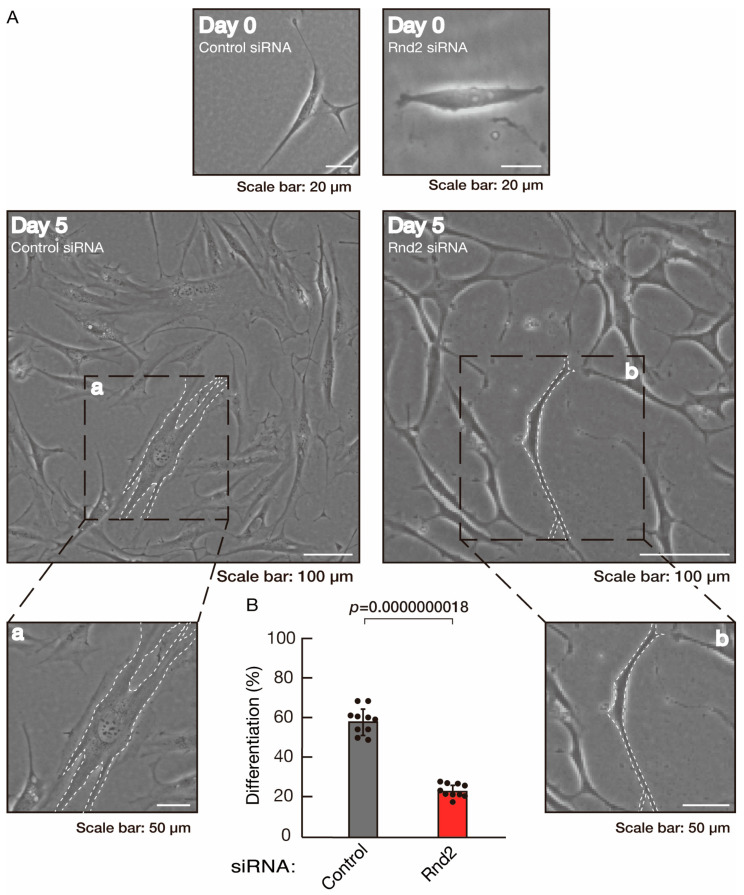
Knockdown of Rnd2 using the RNA interference technique slows morphological differentiation. (**A**,**B**) Cells were transfected with siRNA for control (luciferase) or Rnd2. Following the induction of differentiation, the respective cell morphologies at 0 and 5 days were captured as representative images, analyzed, and statistically depicted (*p* < 0.01; n = 10 fields). Typical cells with widespread membranes are surrounded by dashed lines. The inset images (a,b) are magnified from the squares in the large images.

**Figure 4:**
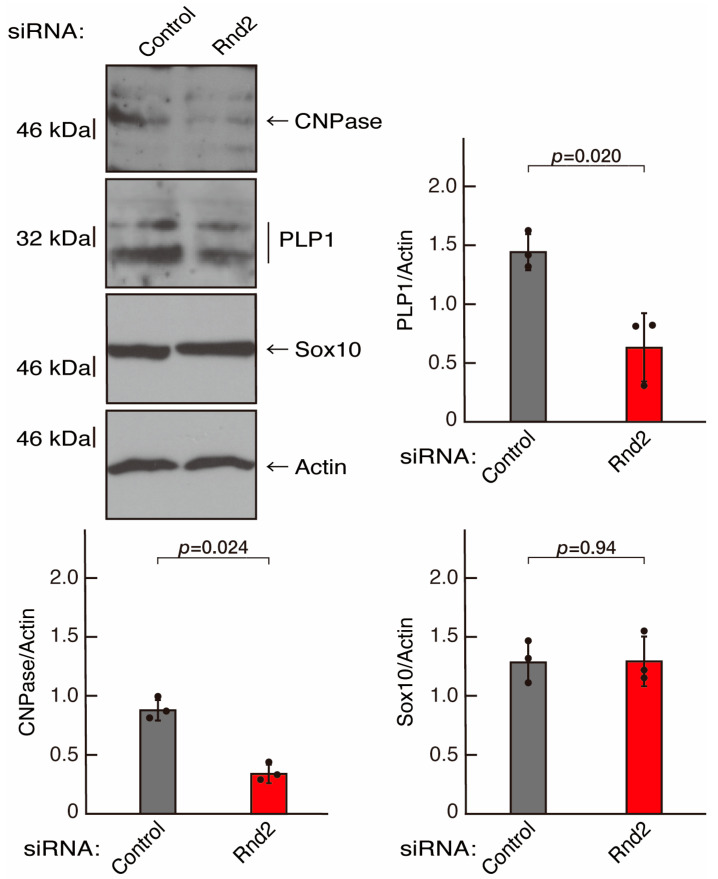
Knockdown of Rnd2 using the RNA interference technique decreases the expression levels of differentiation markers. Cells were transfected with siRNA for control (luciferase) or Rnd2. Following the induction of differentiation, the respective immunoblots at 5 days were captured as the representative images, analyzed, and statistically depicted as immunoreactive bands normalized to actin ones (n = 3 blots).

To further confirm that Rnd2 positively mediates morphological differentiation, we performed a general knockdown technique using siRNA in cells (Figure S2). Transfection of siRNA for Rnd2 but not control (luciferase siRNA) led to retardation of morphological differentiation (Figure 3A,B). The results were consistent with decreased expression of CNPase and PLP1 following the induction of differentiation in Rnd2 knocked-down cells (Figure 4). In contrast, the expression of Sox10 and actin was comparable in control knocked-down and Rnd2-knocked-down cells, indicating that Rnd2 is involved in promoting morphological differentiation.

### 3.2. Rnd2 Effector Molecules Prag1 and Fyn Positively Regulate Morphological Differentiation

**Figure 5:**
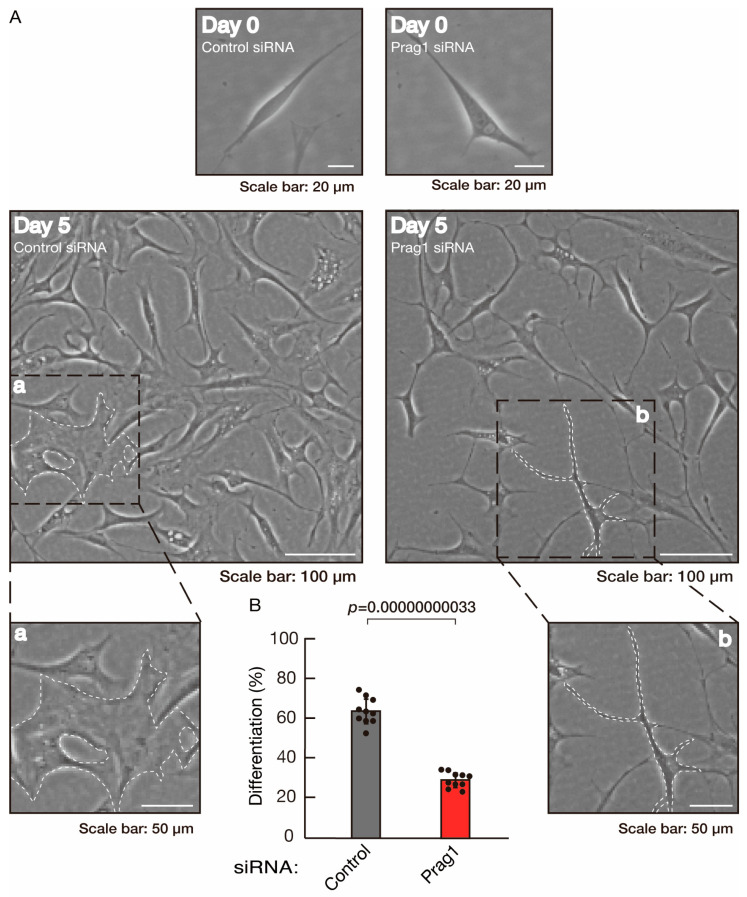
Knockdown of Prag1 slows morphological differentiation. (**A**,**B**) Cells were transfected with siRNA for control or Prag1. Following the induction of differentiation, the respective cell morphologies at 0 and 5 days were captured as the representative images, analyzed, and statistically depicted (*p* < 0.01; n = 10 fields). Typical cells with widespread membranes are surrounded by dashed lines. The inset images (a,b) are magnified from the squares in the large images.

**Figure 6:**
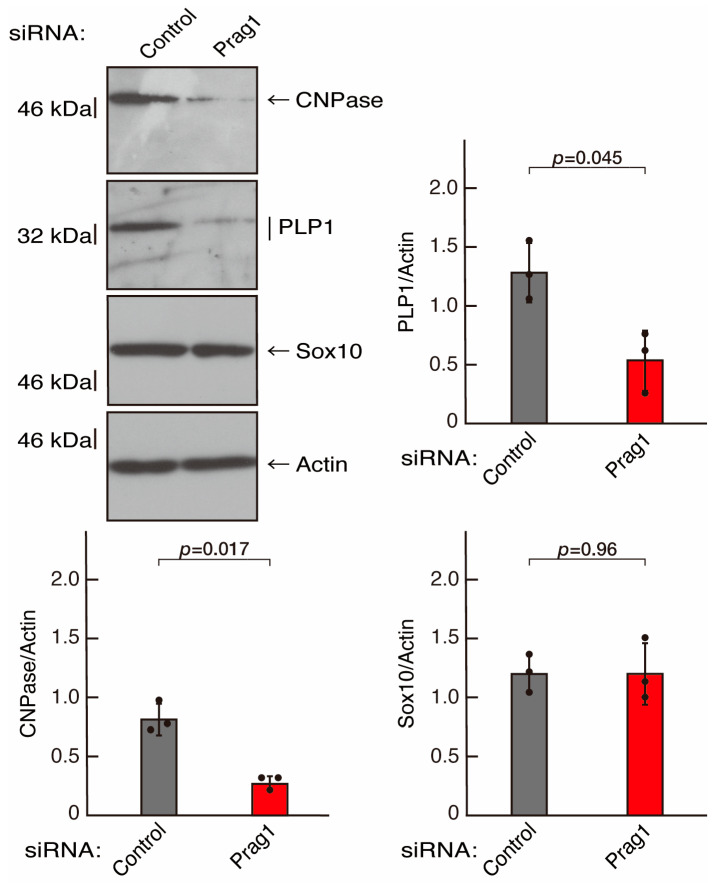
Knockdown of Prag1 decreases the expression levels of differentiation markers. Cells were transfected with siRNA for control or Prag1. Following the induction of differentiation, the respective immunoblots at 5 days were captured as the representative images, analyzed, and statistically depicted as immunoreactive bands normalized to actin ones. (n = 3 blots).

**Figure 7:**
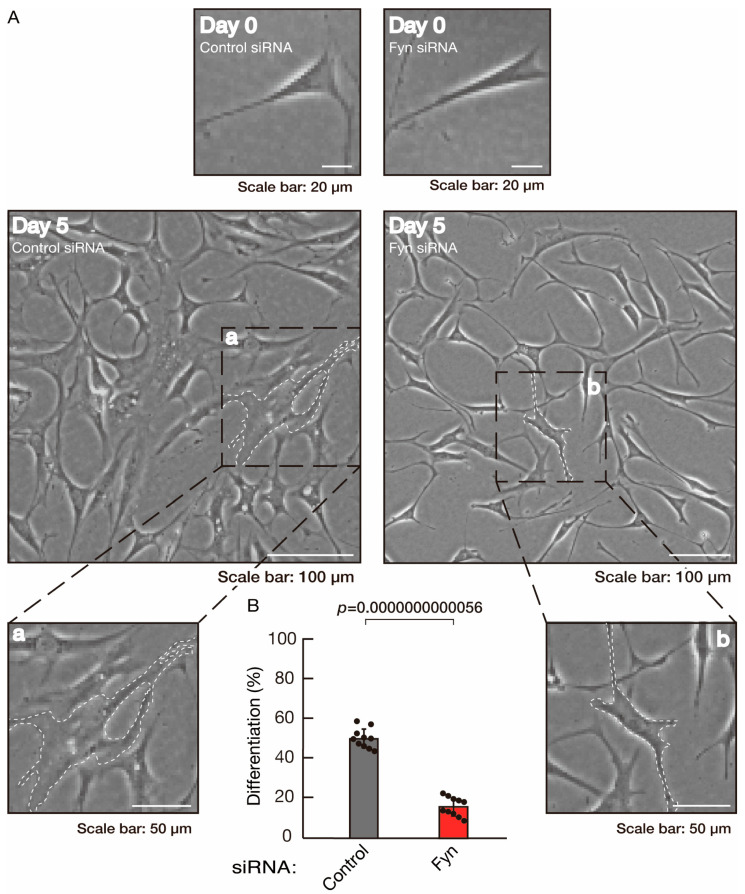
Knockdown of Fyn slows morphological differentiation. (**A**,**B**) Cells were transfected with siRNA for control or Fyn. Following the induction of differentiation, the respective cell morphologies at 0 and 5 days were captured as representative images, analyzed, and statistically depicted (*p* < 0.01; n = 10 fields). Typical cells with widespread membranes are surrounded by dashed lines. The inset images (a,b) are magnified from the squares in the large images.

**Figure 8:**
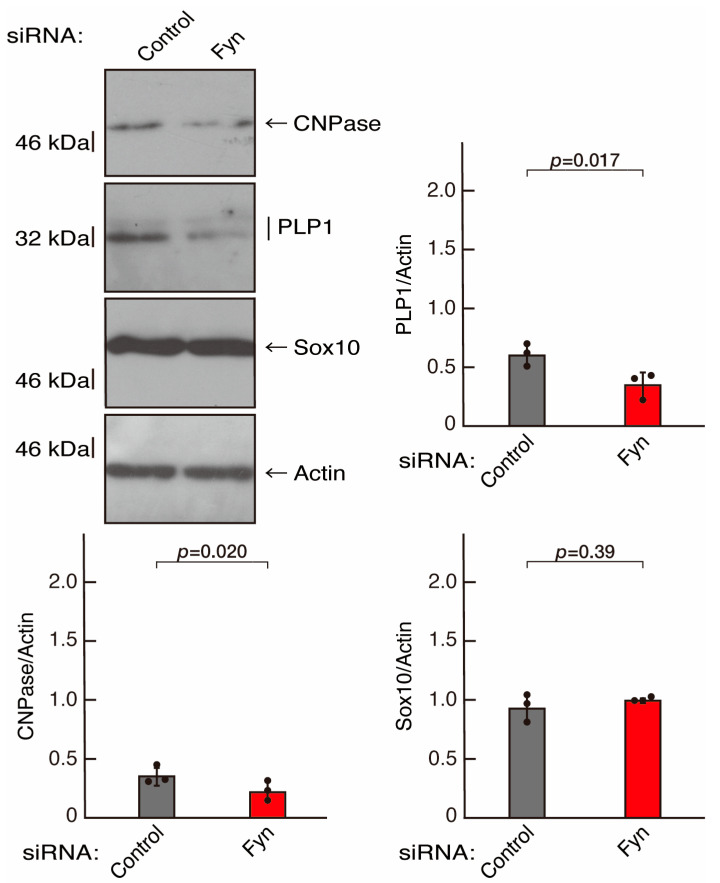
Knockdown of Fyn decreases the expression levels of differentiation markers. Cells were transfected with siRNA for control or Fyn. Following the induction of differentiation, the respective immunoblots at 5 days were captured as the representative images, analyzed, and statistically depicted as immunoreactive bands normalized to actin ones. (n = 3 blots).

To clarify whether the Rnd2 major downstream signaling unit composed of Prag1 (an adaptor protein to block Csk negatively regulating Fyn kinase) [17,18,19,20,35,36] and Fyn kinase (an essential regulator of oligodendroglial cell differentiation and myelination) [17,18,19,20,37,38] is involved in the regulation of oligodendroglial cell morphological differentiation, we first knocked down Prag1 in the FBD-102b cells (Figure S3). As expected, the knockdown of Prag1 led to morphological differentiation retardation (Figure 5A,B). The expression of CNPase and PLP1, but not of Sox10 and actin, was decreased by the knockdown of Prag1 (Figure 6). Similar results were obtained with the knockdown of Fyn (Figure S4 and Figure 7A,B). The two bands observed in the RT-PCR might represent the major long and short forms of Fyn (see the Gene website, https://www.ncbi.nlm.nih.gov/gene/14360, accessed on 1 April 2022). Fyn knockdown also decreased the expression of CNPase and PLP1 but not that of Sox10 and actin (Figure 8). The results of the Parg1 or Fyn knockdown indicate that Prag1 and Fyn, belonging to the Rnd2 signaling cascade, are involved in promoting morphological differentiation.

**Figure 9:**
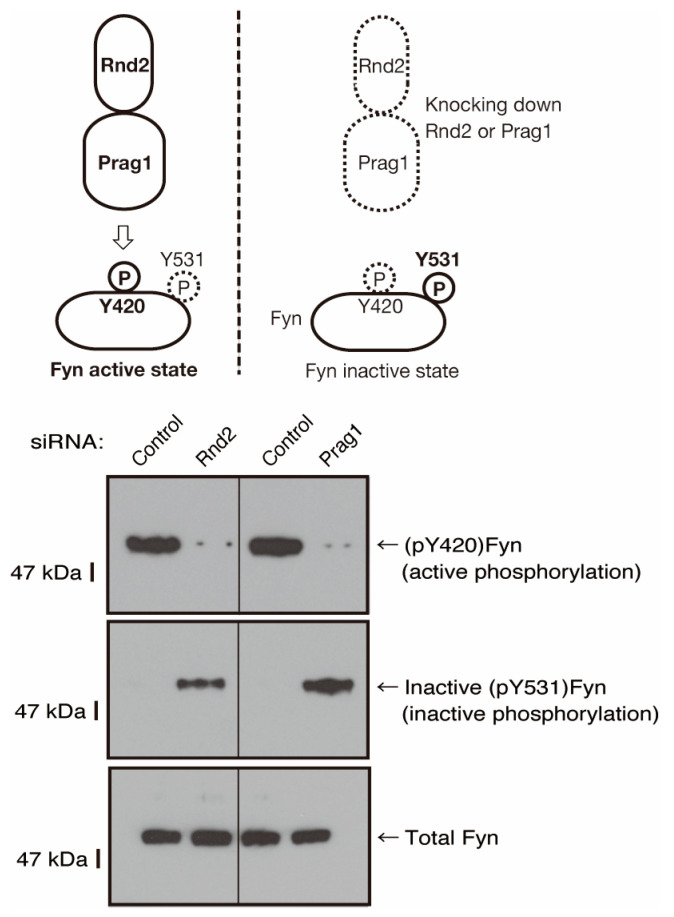
The effects of knockdown of Rnd2 or Parg1 on Fyn phosphorylation states. Cells were transfected with siRNA for control or Rnd2 and also with siRNA for control or Prag1. The respective immunoblots for (pTyr[pY]420)Fyn (active phosphorylation of Y420) or inactive (pY531)Fyn (inactive phosphorylation of Y531) in Fyn immune-precipitates, as well as Fyn (total Fyn), were captured as the representative images of 3 blots.

To explore whether Rnd2 and Prag1 actually mediate the activity of Fyn, we evaluated the phosphorylation levels of both the Fyn autophosphorylation site (Tyr-420) in the kinase activation loop and the C-terminal negative phosphorylating tyrosine (Tyr-531) using negative regulators such as Csk [39,40,41,42]. In Rnd2-knocked-down FBD-102b cells, the phosphorylation of Tyr-420 was decreased, whereas the phosphorylation of Tyr-531 was increased (Figure 9). Similarly, in the Prag1-knocked-down cells, the phosphorylation of Tyr-420 was decreased, whereas the phosphorylation of Tyr-531 was increased, indicating that Fyn can be regulated by Rnd2 and Prag1 in cells. It is thus thought that the Rnd2 and Prag1 signaling unit actually regulates the phosphorylation states of Fyn in FBD-102b cells.

### 3.3. Hesperetin Recovers Phenotypes Induced by the Knockdown of Rnd2/Prag1/Fyn

**Figure 10:**
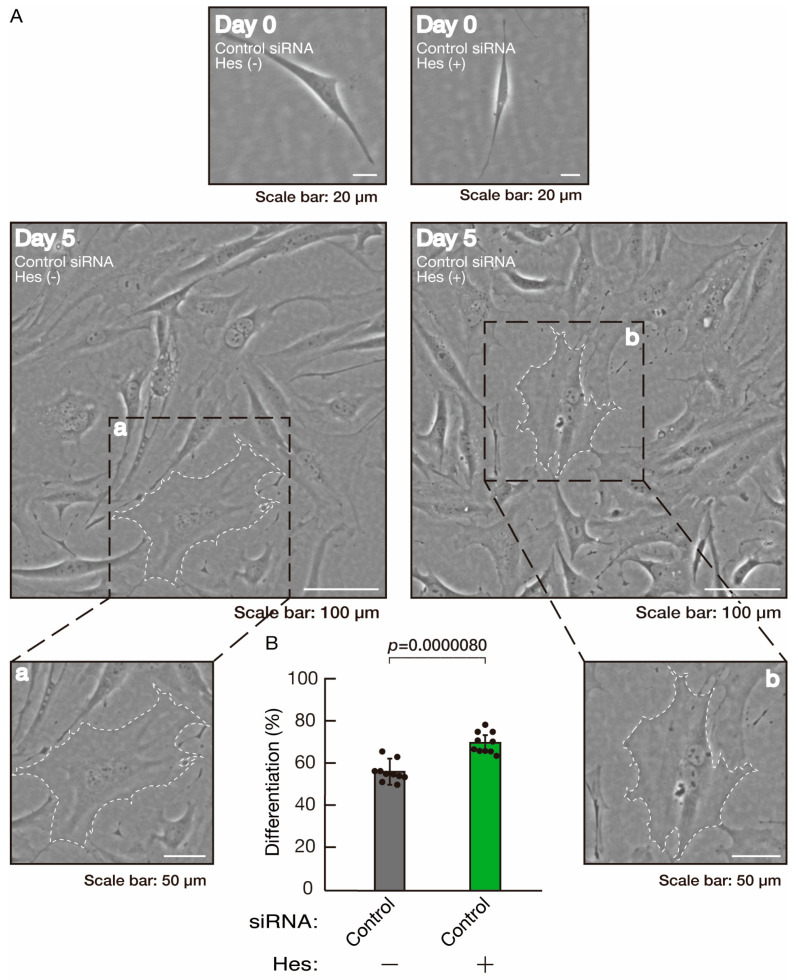
The effects of hesperetin on morphological differentiation in control knockdown conditions. (**A**,**B**) Cells were transfected with siRNA for control (luciferase) in the presence or absence (vehicle control) of hesperetin (Hes). Following the induction of differentiation, the respective cell morphologies at 0 and 5 days were captured as representative images, analyzed, and statistically depicted (*p* < 0.01; n = 10 fields). Typical cells with widespread membranes are surrounded by dashed lines. The inset images (a,b) are magnified from the squares in the large images.

**Figure 11:**
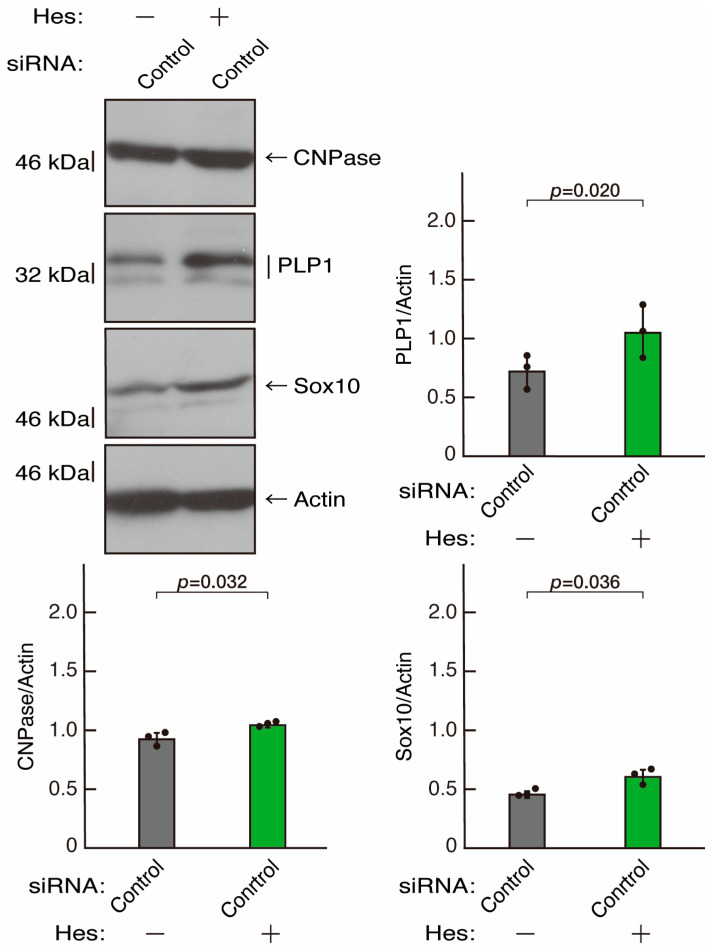
The effects of hesperetin on the expression levels of differentiation markers in control knockdown conditions. Cells were transfected with siRNA for control (luciferase) in the presence or absence (vehicle control) of hesperetin (Hes). Following the induction of differentiation, the respective immunoblots at 5 days were captured as the representative images, analyzed, and statistically depicted as immunoreactive bands normalized to actin ones. (n = 3 blots).

Hesperetin, a citrus flavonoid, has protective effects on both neuronal and glial cells [43,44]. As we previously reported, hesperetin has the ability to recover certain inflammatory cytokine- or disease-related gene-induced retardation of oligodendroglial cell-like morphological differentiation [45,46]. First, to confirm that hesperetin can induce morphological differentiation in control knockdown experiments, we transfected control siRNA into cells and treated them with hesperetin or a control vehicle. Hesperetin increased morphological differentiation (Figure 10A,B), a finding supported by the increased expression of CNPase and PLP1 (Figure 11). Since the expression of Sox10 was also increased, hesperetin may have a protective effect. In contrast, the expression of actin was comparable in the presence or absence of hesperetin, suggesting that hesperetin is weakly but significantly involved in promoting differentiation.

We further examined whether monoglucosyl hesperidine, a highly soluble hesperetin derivative with in vivo protective effects as well as cellular effects [47,48], has similar effects to hesperetin. Treatment with monoglucosyl hesperidine promoted differentiation in control siRNA-transfected cells, as seen following treatment with hesperetin (Figure S5). These effects were supported by the increased expression of CNPase and PLP1, but not of Sox10 and actin, following treatment with monoglucosyl hesperidine (Figure S6). These findings suggest that the hesperetin chemical backbone itself has the ability to help promote differentiation.

**Figure 12:**
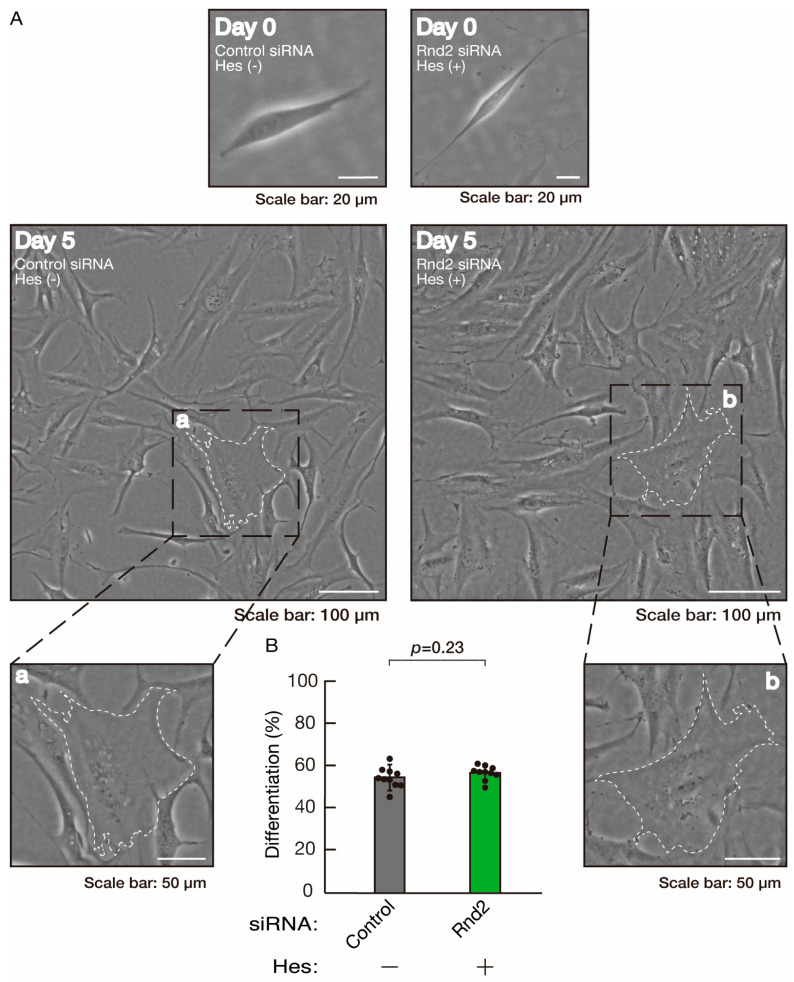
Hesperetin recovers morphological differentiation retardation by knockdown of Rnd2. (**A**,**B**) Cells were transfected with siRNA for control or Rnd2 in the presence or absence of hesperetin (Hes). Following the induction of differentiation, the respective cell morphologies at 0 and 5 days were captured as representative images, analyzed, and statistically depicted (n = 10 fields). Typical cells with widespread membranes are surrounded by dashed lines. The inset images (a,b) are magnified from the squares in the large images.

**Figure 13:**
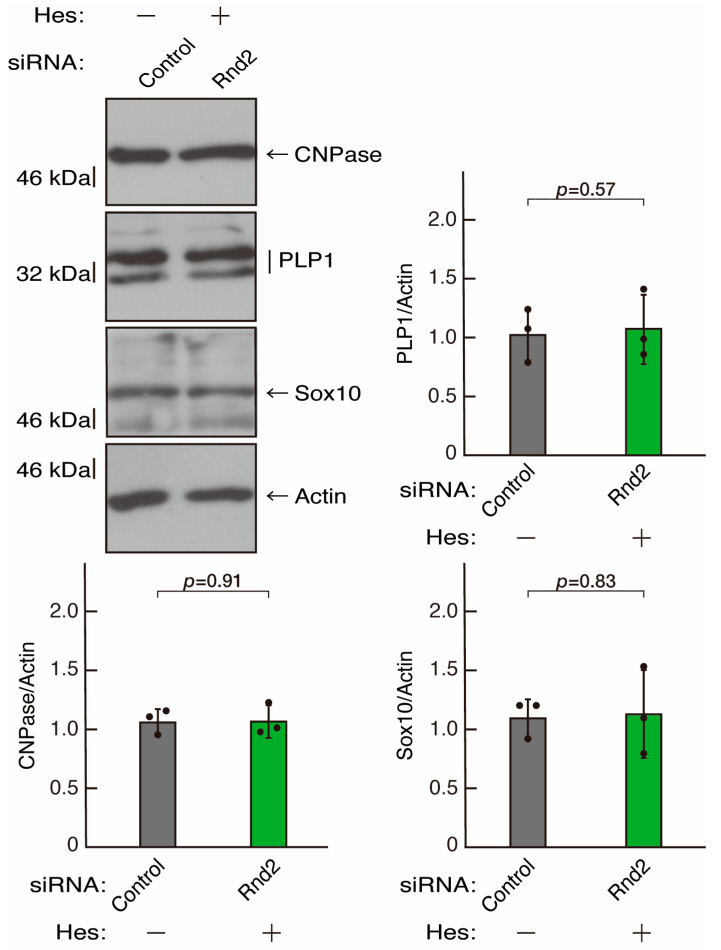
Hesperetin recovers decreased expression levels of differentiation markers by knockdown of Rnd2. Cells were transfected with siRNA for control or Rnd2 in the presence or absence of hesperetin (Hes). Following the induction of differentiation, the respective immunoblots at 5 days were captured as the representative images, analyzed, and statistically depicted as immunoreactive bands normalized to actin ones (n = 3 blots).

**Figure 14:**
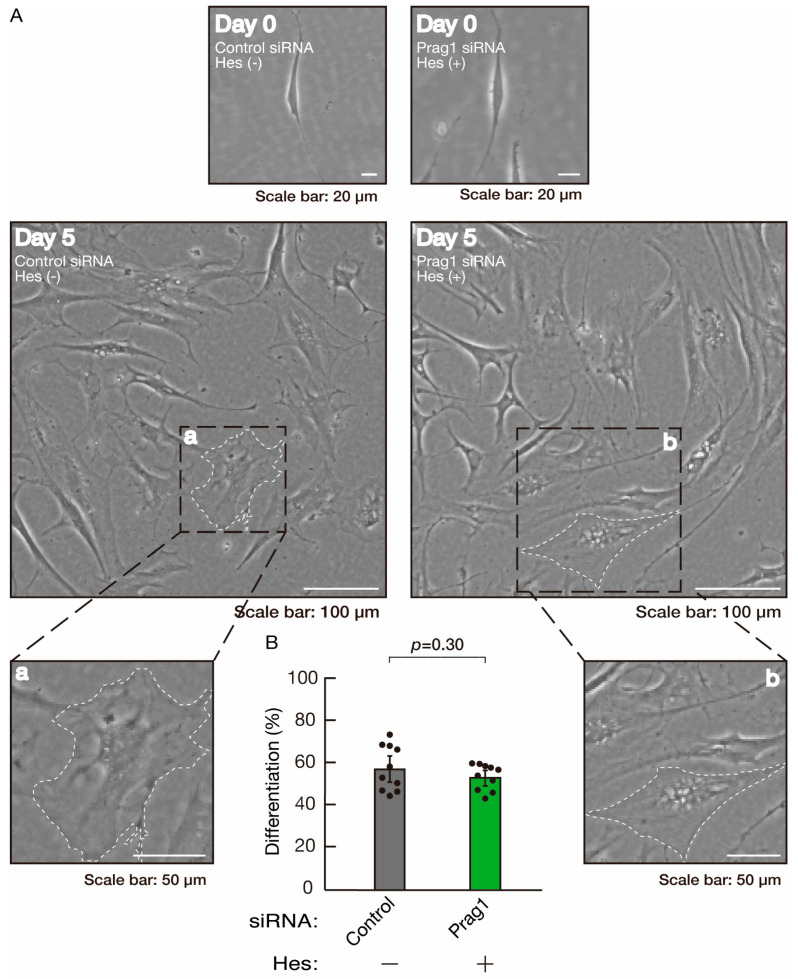
Hesperetin recovers morphological differentiation retardation by knockdown of Prag1. (**A**,**B**) Cells were transfected with siRNA for control or Prag1 in the presence or absence of hesperetin (Hes). Following the induction of differentiation, the respective cell morphologies at 0 and 5 days were captured as representative images, analyzed, and statistically depicted (n = 10 fields). Typical cells with widespread membranes are surrounded by dashed lines. The inset images (a,b) are magnified from the squares in the large images.

**Figure 15:**
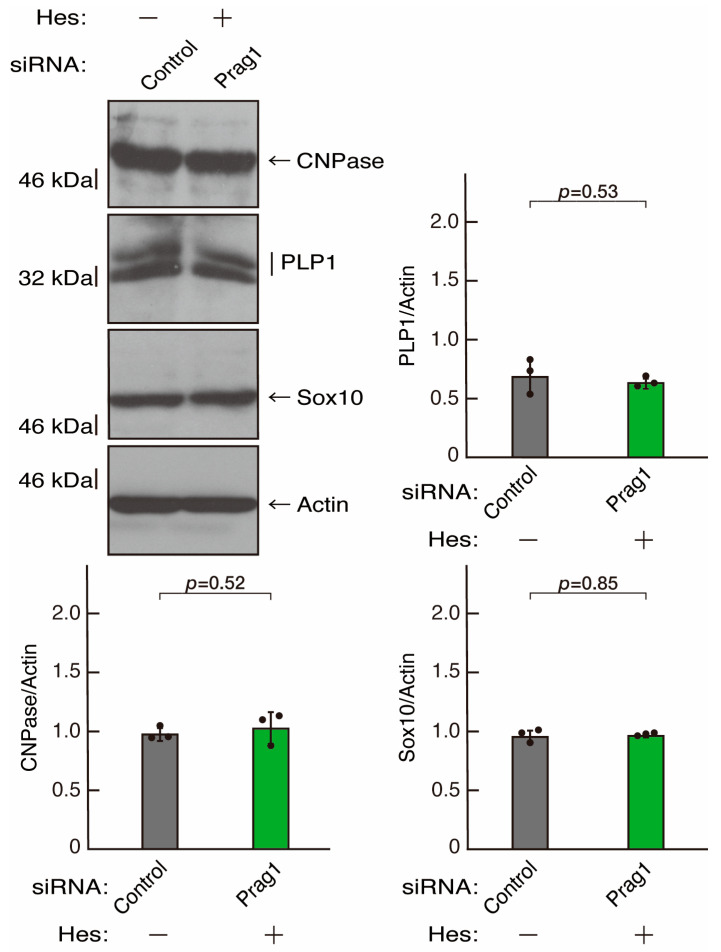
Hesperetin recovers decreased expression levels of differentiation markers by knockdown of Prag1. Cells were transfected with siRNA for control or Prag1 in the presence or absence of hesperetin (Hes). Following the induction of differentiation, the respective immunoblots at 5 days were captured as the representative images, analyzed, and statistically depicted as immunoreactive bands normalized to actin ones (n = 3 blots).

**Figure 16:**
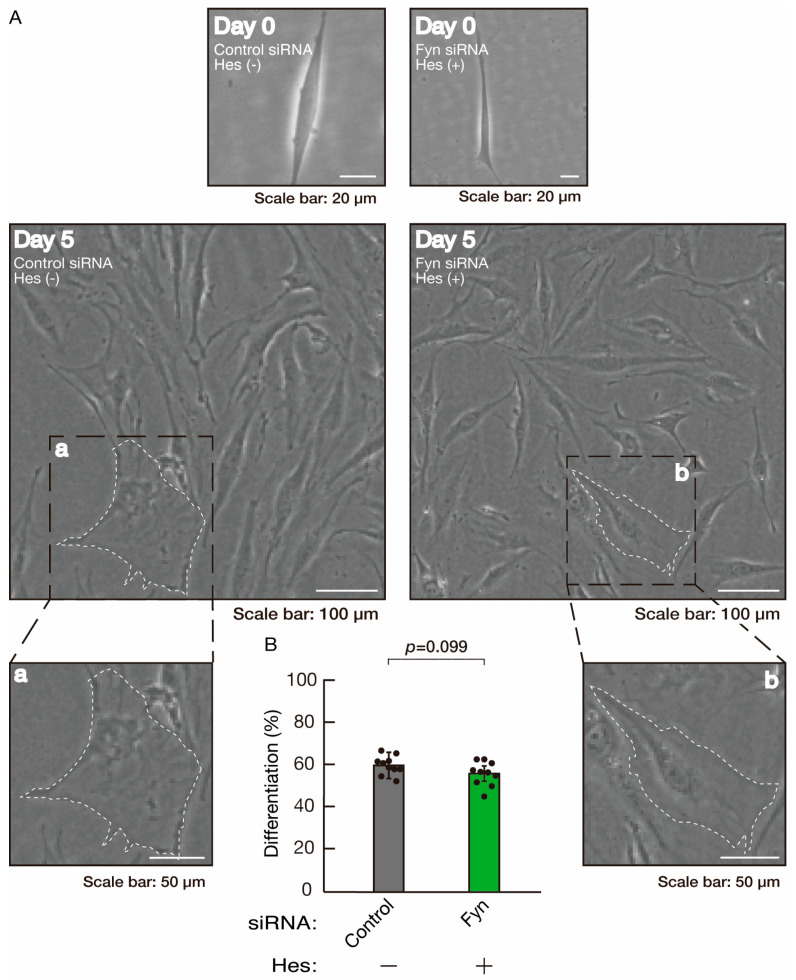
Hesperetin recovers retardation of morphological differentiation by knockdown of Fyn. (**A**,**B**) Cells were transfected with siRNA for control or Fyn in the presence or absence of hesperetin (Hes). Following the induction of differentiation, the respective cell morphologies at 0 and 5 days were captured as representative images, analyzed, and statistically depicted (n = 10 fields). Typical cells with widespread membranes are surrounded by dashed lines. The inset images (a,b) are magnified from the squares in the large images.

**Figure 17:**
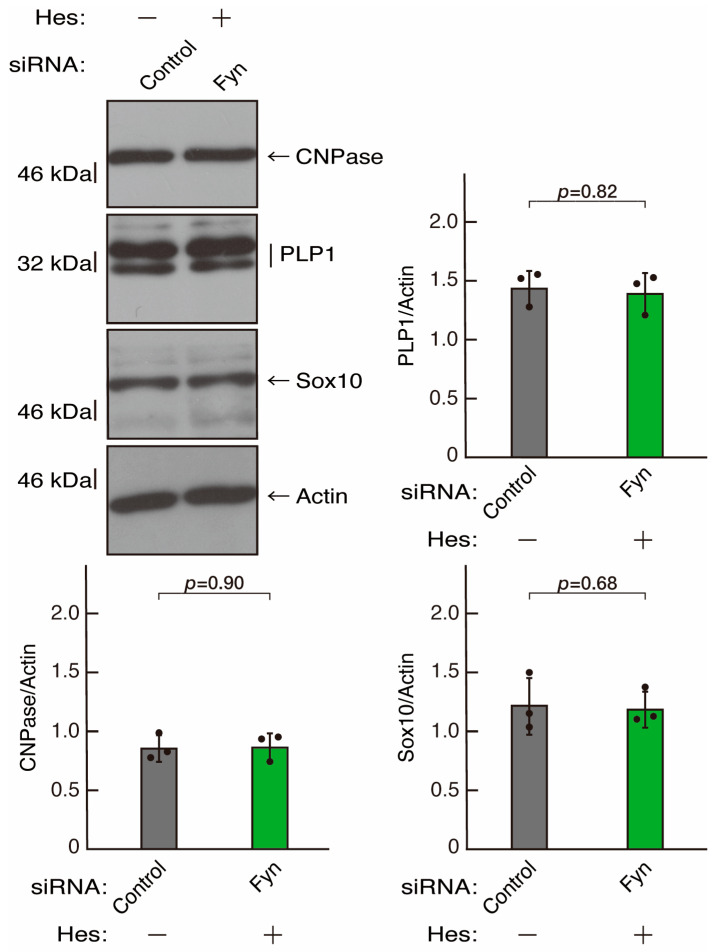
Hesperetin recovers decreased expression levels of differentiation markers by knockdown of Fyn. Cells were transfected with siRNA for control or Fyn in the presence or absence of hesperetin (Hes). Following the induction of differentiation, the respective immunoblots at 5 days were captured as the representative images, analyzed, and statistically depicted as immunoreactive bands normalized to actin ones. (n = 3 blots).

We next determined whether hesperetin could recover retardation of oligodendroglial cell-like morphological differentiation by knockdown of Rnd2. Of note, hesperetin recovered phenotypes induced by Rnd2 knockdown at the same level as control knockdown (Figure 12A,B). Consistently, the decreased expression of CNPase and PLP1 induced by Rnd2 knockdown was recovered by hesperetin at the same level as the control knockdown (Figure 13). Similar results were obtained in the case of Prag1 (Figure 14A,B and Figure 15) and Fyn (Figure 16A,B and Figure 17), indicating that hesperetin can recover retardation of morphological differentiation by the knockdown of Rnd2/Preg1/Fyn.

### 3.4. Results Obtained from This Study

Knockdown using siRNA and/or gRNA against Rnd2 or the downstream signaling molecules Prag1 or Fyn indicates that the knockdown of each molecule reduces the ability of the FBD-102b cells to differentiate morphologically. These results are accompanied by decreased levels of differentiation markers and altered levels of Fyn phosphorylation. In contrast, treatment with hesperetin recovers these knockdown effects.

## 4. Discussion

In the present study, we showed that Rnd2 promoted oligodendroglial cell-like morphological differentiation in an FBD-102b cell line. The knockdown of Rnd2 using the specific gRNA-based RNA editing or siRNA technique leads to retardation of morphological differentiation. Similarly, the knockdown of the downstream effector cascade molecule Prag1 or Fyn also affects differentiation, revealing the positive role of the signaling pathway coupling Rnd2 to Prag1 and Fyn. On the other hand, hesperetin recovers retardation of morphological differentiation by each molecular knockdown.

Oligodendroglial cell differentiation is a preparatory process for myelination [17,18,19,20]. Thus, the signal linking Rnd2 to Prag1 and Fyn may be preserved during myelination. In addition, Rnd2 has dual roles depending on the developmental period in myelination by oligodendroglial cells; Rnd2 acts as a positive regulator during the early myelination period and as a negative regulator during the late developing period [25]. Our results are consistent with the positive role of Rnd2 in the early period.

All Rnd proteins (Rnd1, Rnd2, and Rnd3) are widely expressed throughout tissues and are unusual in that they bind to GTP but are almost incapable of hydrolysis of GTP [13,14,15,16]. Unlike other small GTPases, Rnd proteins are not thought to be regulated by conformational switches for GTP- and GDP-binding forms. The activities of Rnd proteins are primarily regulated by expression levels during development [13,14,15,16]. Because transfection of the Rnd proteins is known to change the formation of actin stress fibers in fibroblasts and epithelial cells, the Rnd proteins often act antagonistically to RhoA, the actin cytoskeletal regulator, by modulating the downstream signaling of RhoA [13,14,15,16]. RhoA and downstream signaling promote stress fiber formation, specifically inhibiting myelination following differentiation in oligodendroglial cells [49]. It is therefore presumed that Rnd proteins promote myelination. Research groups, including our own, report that Rnd2 and Rnd3 are involved in the regulation of myelination, at least in part [25,50]. Despite the increased expression of Rnd2 protein [25], it is unclear whether Rnd3 and Rnd1 are upregulated as brain development proceeds [50]. It is thought that the activities of Rnd3 and probably Rnd1, acting cooperatively with those of Rnd2, are needed to trigger myelination.

In contrast, the functional loss of Rnd2 and the possible effector signaling molecules underlying Rnd2 are related to some diseases including neuropsychiatric disorders, which are often associated with abnormalities of oligodendroglial differentiation and myelination and neuronal differentiation [6,7,8]. Rnd2 is weakly but specifically expressed in cortical plates in embryos and in some neuronal cells of the hippocampal regions in adults [51,52]. It is conceivable that neuronal cells act cooperatively with oligodendroglial precursor cells or oligodendroglial cells to generate specific brain regions such as the hippocampus.

We failed to observe the negative roles of Rnd2 and the effector molecules in morphological differentiation in the FBD-102b cells, as seen in the late developing period [25]. This might be because the in vitro systems are not suitable for long-term observations. Thus, the experiment ended before the negative effects of Rnd2 became apparent in the cells. Alternatively, signaling units composed of Rnd2 and Rnd2 effector molecules may differ depending on the respective developmental periods: an early period centered on cell differentiation and a late period centered on dynamic morphological changes such as myelination. In either case, it is clear that molecules associated with signaling through Rnd2 can be involved in fine-tuning morphogenesis within oligodendroglial cells [25].

Prag1 was originally identified as an actin cytoskeletal regulatory protein with the ability to interact with Rnd2 [15]. Additionally, at present, Prag1 is known to be able to sequester Csk as an inhibitor of Fyn [17,18,19,20,35,36], resulting in stimulating the tyrosine kinase activity of Fyn, the master regulator of oligodendroglial cell morphogenesis [17,18,19,20,37,38]. There is increasing evidence that Fyn has many biochemically identified substrates, including a variety of cytoskeletal and structural proteins [53,54]. Fyn may contribute to cytoskeletal changes to trigger morphogenesis in oligodendroglial cells. Furthermore, according to the BioGRID website (https://thebiogrid.org/, accessed on 1 April 2022), Prag1 has some potential interaction proteins, including cytoskeletal and structural proteins. Prag1, acting downstream of Rnd2, may regulate actin cytoskeletal proteins to modulate morphological differentiation. It is thus suggested that Prag1 or Fyn, or both, regulate cellular morphogenesis through more complicated molecular mechanisms than previously thought.

Flavonoids, including hesperidin and its aglycon hesperetin, are known to decrease the neuroinflammation involved in the progression of neurodegenerative disorders such as Alzheimer’s disease, Parkinson’s disease, amyotrophic lateral sclerosis, and multiple sclerosis [43,44]. Hesperidin and hesperetin could also delay the progression of CNS neuropathies. Although it is unlikely that flavonoids such as hesperetin can block the degeneration of neuronal and glial cells, hesperetin can modulate the activities of some signaling molecules [43,44]. For example, hesperetin binds to tyrosine phosphatase 1B (PTP1B) with wide substrate specificities [55]. PTP1B is known to be a negative regulator of signaling molecules around tyrosine-phosphorylated insulin receptor substrate 1 (IRS1) and the downstream Akt kinase in oligodendroglial cells [56,57,58]. Signaling through Akt plays a key role in oligodendroglial cell differentiation and myelination [56,57,58]. It is conceivable that modulation of PTP1B activities by hesperetin, acting through Akt signaling, can affect them; however, it remains unclear whether hesperetin inhibits the activities of PTP1B in oligodendroglial cells. Furthermore, if the relationship between hesperetin and dephosphorylating enzymes is a general molecular mechanism [59,60], hesperetin might promote morphological differentiation by inhibiting the dephosphorylation of the Fyn substrates under some pathological conditions.

This study has five main limitations: (1) The oligodendrocyte cell line FBD-102b was used instead of primary cultured cells. Although FBD-102b cells have differentiation potential, they preserve only some of the properties of the primary cells; thus, care must be taken when interpreting results from the cell line. One possible solution might be to conduct several similar experiments to support one conclusion. Further studies using primary cells are required to interpret the results obtained from this study; (2) Established cell lines with differentiation potential are generally immortalized and proliferative. It is often difficult to observe cell lines for long periods during an experiment in the differentiation phase beyond the proliferation phase. Unlike primary cells, cell lines differentiate slowly. Observation after several days indicates that knockdown results in a slowing of differentiation, rather than complete inhibition; (3) At day 0, the FBD-102b cells display spindle-like phenotypes, as seen in primary oligodendroglial precursor cells [26,27], and cell morphologies at day 0 are similar regardless of control- and target signaling molecule-siRNA or gRNA transfection. As the experiment proceeds following the induction of differentiation, the cells exhibit oligodendroglial cell-like differentiated morphology, in which branches extend from the cell body and the cytoplasmic regions slowly expand [45,46]. Differentiation retardation occurs only in the target signaling molecule knockdown cells, not the control knockdown cells. Only at the final time point are the effects of marker protein expression observed. However, unlike primary oligodendroglial precursor cells, FBD-102b cells at day 0 express some differentiation marker proteins at low levels [26,27]. Again, caution is warranted when interpreting results from the cell line; (4) In the immunoblotting studies, several sets of experiments were performed. For all sets of scanned immunoreactive protein band values, comparisons between all groups were performed using data values normalized to actin band ones; (5) Because our results were not obtained from genetically modified mice, they cannot be directly compared to previous results using genetically modified mice [25].

Further studies are needed to increase our understanding of the underlying neuropsychiatric disorder-related Rnd2 molecular mechanisms. Studies may elucidate not only how Rnd2/Prag1/Fyn signaling is required for oligodendroglial cell morphological differentiation and myelination but also how Rnd2/Prag1/Fyn signaling oligodendroglial cell differentiation is cooperatively linked with neuronal cells at the molecular and cellular levels. Additionally, these studies could lead to a better understanding of which signaling pathway is affected by hesperetin in oligodendroglial cells in vivo as well as in vivo. Studies in this direction might allow us to develop target-specific medicines for oligodendroglial cell-related neuropsychiatric disorders [61,62,63,64].

## Figures and Tables

**Table 1:** Key antibodies and chemicals used in the study.

Reagents or Sources	Company or Source	Cat. No.	Lot. No.	Concentration Used
Antibodies				
Anti-proteolipid protein 1 (PLP1)	Atlas Antibodies	HPA004128	8115828	Immunoblotting (IB), 1:500
Anti-2′,3′-cyclic nucleotides to 2′-nucleotides (CNPase)	Santa Cruz Biotechnology	sc-166559	A1514	IB, 1:500
Anti-Sox10	Santa Cruz Biotechnology	sc-365692	F1621	IB, 1:500
Anti-actin (also called pan-b type actin)	MBL	M177-3	007	IB, 1:5000
anti-Fyn	Atlas Antibodies	HPA023887	A75443	IB, 1:500; immunopresipitation (IP), 0.1 mg for 300 mg proteins of the respective cell lysates
anti-p-c-Src (9A6), which corresponds to anti-(Py420)Fyn	Santa Cruz Biotechnology	sc-81521	F2122	IB, 1:100
anti-p-c-Src (H3), which corresponds to anti-(Py531)Fyn	Santa Cruz Biotechnology	sc-166860	I2921	IB, 1:100
Anti-IgG (H+L chain) (Rabbit) pAb-HRP	MBL	458	353	IB, 1:5000
Anti-IgG (H+L chain) (Mouse) pAb-HRP	MBL	330	365	IB, 1:5000
Key chemicals				
Hesperetin (Hes)	FUJIFILM Wako Pure Chemical Corporation	087-10001	DLK5755	15 Mm (final concentration)
Monoglucosyl hesperidine (M.Hes)	FUJIFILM Wako Pure Chemical Corporation-Hayashibara Co., Ltd.	638-07361	191127	25 Mm (final concentration)
Dimethyl sulfoxide (DMSO)	FUJIFILM Wako Pure Chemical Corporation	047-29353	CDN0170	Less than 0.1% (final concentration)

## Data Availability

The datasets used and/or analyzed for the current study are available from the corresponding author upon reasonable request.

## Supplementary Materials

The following supporting information can be downloaded at: https://www.mdpi.com/article/10.3390/neurolint16010003/s1, Figure S1. Effects of knockdown of Rnd2 using the CRISPR/CasRx system; Figure S2. Effects of knockdown of Rnd2 using the RNA interference technique; Figure S3. Effects of knockdown of Prag1 using the RNA interference technique; Figure S4. Effects of knockdown of Fyn using the RNA interference technique; Figure S5. Effects of monoglucosyl hesperidine on morphological differentiation in control knockdown conditions; Figure S6. Effects of monoglucosyl hesperidine on the expression levels of differentiation markers in control knockdown conditions; Figure S7. Original size gels of Figure 2; Figure S8. Original size gels of Figure 4; Figure S9. Original size gels of Figure 6; Figure S10. Original size gels of Figure 8; Figure S11. Original size gels of Figure 9; Figure S12. Original size gels of Figure 11; Figure S13. Original size gels of Figure 13; Figure S14. Original size gels of Figure 15; Figure S15. Original size gels of Figure 17; Figure S16. Original size gels of Figure S1; Figure S17. Original size gels of Figure S2; Figure S18. Original size gels of Figure S3; Figure S19. Original size gels of Figure S4; and Figure S20. Original size gels of Figure S6. Table S1. Statistical data for Figure 1; Table S2. Statistical data for Figure 2; Table S3. Statistical data for Figure 3; Table S4. Statistical data for Figure 4; Table S5. Statistical data for Figure 5; Table S6. Statistical data for Figure 6; Table S7. Statistical data for Figure 7; Table S8. Statistical data for Figure 8; Table S9. Statistical data for Figure 10; Table S10. Statistical data for Figure 11; Table S11. Statistical data for Figure 12; Table S12. Statistical data for Figure 13; Table S13. Statistical data for Figure 14; Table S14. Statistical data for Figure 15; Table S15. Statistical data for Figure 16; Table S16. Statistical data for Figure 17; Table S17. Statistical data for Figure S5; Table S18. Statistical data for Figure S6.

## References

[1] Agarwal D., Sandor C., Volpato V., Caffrey T.M., Monzón-Sandoval J., Bowden R., Alegre-Abarrategui J., Wade-Martins R., Webber C. (2020). A single-cell atlas of the human substantia nigra reveals cell-specific pathways associated with neurological disorders. Nat. Commun..

[2] Charvériat M., Mouthon F., Rein W., Verkhratsky A. (2021). Connexins as therapeutic targets in neurological and neuropsychiatric disorders. Biochim. Biophys. Acta Mol. Basis Dis..

[3] Akay L.A., Effenberger A.H., Tsai L.H. (2021). Cell of all trades: Oligodendrocyte precursor cells in synaptic, vascular, and immune function. Genes Dev..

[4] Wheeler N.A., Fuss B. (2016). Extracellular cues influencing oligodendrocyte differentiation and (re)myelination. Exp. Neurol..

[5] Decourt B., Bouleau Y., Dulon D., Hafidi A. (2005). Expression analysis of neuroleukin, calmodulin, cortactin, and Rho7/Rnd2 in the intact and injured mouse brain. Dev. Brain Res..

[6] Basbous S., Azzarelli R., Pacary E., Moreau V. (2021). Pathophysiological functions of Rnd proteins. Small GTPases.

[7] Ohtaka-Maruyama C., Hirai S., Miwa A., Heng J.I., Shitara H., Ishii R., Taya C., Kawano H., Kasai M., Nakajima K. (2013). RP58 regulates the multipolar-bipolar transition of newborn neurons in the developing cerebral cortex. Cell Rep..

[8] Kerloch T., Farrugia F., Bouit L., Maître M., Terral G., Koehl M., Mortessagne P., Heng J.I., Blanchard M., Doat H. (2021). The atypical Rho GTPase Rnd2 is critical for dentate granule neuron development and anxiety-like behavior during adult but not neonatal neurogenesis. Mol. Psychiatry.

[9] Bar-Sagi D. (2001). A Ras by any other name. Mol. Cell. Biol..

[10] Hall A. (2005). Rho GTPases and the control of cell behaviour. Biochem. Soc. Trans..

[11] Dahmene M., Quirion L., Laurin M. (2020). High throughput strategies aimed at closing the GAP in our knowledge of Rho GTPase signaling. Cells.

[12] Rossman K.L., Der C.J., Sondek J. (2005). GEF means go: Turning on RHO GTPases with guanine nucleotide-exchange factors. Nat. Rev. Mol. Cell Biol..

[13] Riento K., Villalonga P., Garg R., Ridley A. (2005). Function and regulation of RhoE. Biochem. Soc. Trans..

[14] Chardin P. (2006). Function and regulation of Rnd proteins. Nat. Rev. Mol. Cell Biol..

[15] Tanaka H., Katoh H., Negishi M. (2006). Pragmin, a novel effector of Rnd2 GTPase, stimulates RhoA activity. J. Biol. Chem..

[16] Goh L.L., Manser E. (2012). The GTPase-deficient Rnd proteins are stabilized by their effectors. J. Biol. Chem..

[17] Bergles D.E., Richardson W.D. (2015). Oligodendrocyte development and plasticity. Cold Spring Harb. Perspect. Biol..

[18] Simons M., Nave K.A. (2015). Oligodendrocytes: Myelination and axonal support. Cold Spring Harb. Perspect. Biol..

[19] Tiane A., Schepers M., Rombaut B., Hupperts R., Prickaerts J., Hellings N., van den Hove D., Vanmierlo T. (2019). From OPC to oligodendrocyte: An epigenetic journey. Cells.

[20] Kuhn S., Gritti L., Crooks D., Dombrowski Y. (2019). Oligodendrocytes in development, myelin generation and beyond. Cells.

[21] Barateiro A., Brites D., Fernandes A. (2016). Oligodendrocyte development and myelination in neurodevelopment: Molecular mechanisms in health and disease. Curr. Pharm. Des..

[22] Dulamea A.O. (2017). Role of oligodendrocyte dysfunction in demyelination, remyelination and neurodegeneration in multiple sclerosis. Adv. Exp. Med. Biol..

[23] Zhou B., Zhu Z., Ransom B.R., Tong X. (2021). Oligodendrocyte lineage cells and depression. Mol. Psychiatry.

[24] Clayton B.L.L., Tesar P.J. (2021). Oligodendrocyte progenitor cell fate and function in development and disease. Curr. Opin. Cell Biol..

[25] Miyamoto Y., Torii T., Terao M., Takada S., Tanoue A., Katoh H., Yamauchi J. (2021). Rnd2 differentially regulates oligodendrocyte myelination at different developmental periods. Mol. Biol. Cell.

[26] Horiuchi M., Tomooka Y. (2006). An oligodendroglial progenitor cell line FBD-102b possibly secretes a radial glia-inducing factor. Neurosci. Res..

[27] Okada A., Tomooka Y. (2013). A role of Sema6A expressed in oligodendrocyte precursor cells. Neurosci. Lett..

[28] De Vries G.H., Boullerne A.I. (2010). Glial cell lines: An overview. Neurochem. Res..

[29] Tontsch U., Archer D.R., Dubois-Dalcq M., Duncan I.D. (1994). Transplantation of an oligodendrocyte cell line leading to extensive myelination. Proc. Natl. Acad. Sci. USA.

[30] Sato N., Seiwa C., Uruse M., Yamamoto M., Tanaka K., Kawakita T., Komatsu Y., Yasukawa A., Takao M., Kudo C. (2011). Administration of chinpi, a component of the herbal medicine ninjin-youei-to, reverses age-induced demyelination. Evid. Based Complement Alternat. Med..

[31] Kato Y., Tago K., Fukatsu S., Okabe M., Shirai R., Oizumi H., Ohbuchi K., Yamamoto M., Mizoguchi K., Miyamoto Y. (2022). CRISPR/CasRx-mediated RNA knockdown reveals that ACE2 is involved in the regulation of oligodendroglial cell morphological differentiation. Noncoding RNA.

[32] Tago K., Kaziro Y., Satoh T. (1998). Functional involvement of mSos in interleukin-3 and thrombin stimulation of the Ras, mitogen-activated protein kinase pathway in BaF3 murine hematopoietic cells. J. Biochem..

[33] Konermann S., Lotfy P., Brideau N.J., Oki J., Shokhirev M.N., Hsu P.D. (2018). Transcriptome engineering with RNA-targeting type VI-D CRISPR effectors. Cell.

[34] Yan W.X., Chong S., Zhang H., Makarova K.S., Koonin E.V., Cheng D.R., Scott D.A. (2018). Cas13d Is a compact RNA-targeting type VI CRISPR effector positively modulated by a WYL-domain-containing accessory protein. Mol. Cell.

[35] Safari F., Murata-Kamiya N., Saito Y., Hatakeyama M. (2011). Mammalian pragmin regulates Src family kinases via the Glu-Pro-Ile-Tyr-Ala (EPIYA) motif that is exploited by bacterial effectors. Proc. Natl. Acad. Sci. USA.

[36] Senda Y., Murata-Kamiya N., Hatakeyama M. (2016). C-terminal Src kinase-mediated EPIYA phosphorylation of Pragmin creates a feed-forward C-terminal Src kinase activation loop that promotes cell motility. Cancer Sci..

[37] Umemori H., Sato S., Yagi T., Aizawa S., Yamamoto T. (1994). Initial events of myelination involve Fyn tyrosine kinase signalling. Nature.

[38] Matrone C., Petrillo F., Nasso R., Ferretti G. (2020). Fyn tyrosine kinase as harmonizing factor in neuronal functions and dysfunctions. Int. J. Mol. Sci..

[39] Roskoski R. (2004). Src protein-tyrosine kinase structure and regulation. Biochem. Biophys. Res. Commun..

[40] Okada M. (2012). Regulation of the SRC family kinases by Csk. Int. J. Biol. Sci..

[41] Roskoski R. (2015). Src protein-tyrosine kinase structure, mechanism, and small molecule inhibitors. Pharmacol. Res..

[42] Zhu S., Wang H., Ranjan K., Zhang D. (2023). Regulation, targets and functions of CSK. Front. Cell Dev. Biol..

[43] Spagnuolo C., Moccia S., Russo G.L. (2018). Anti-inflammatory effects of flavonoids in neurodegenerative disorders. Eur. J. Med. Chem..

[44] Bandiwadekar A., Jose J., Khayatkashani M., Habtemariam S., Khayat Kashani H.R., Nabavi S.M. (2022). Emerging novel approaches for the enhanced delivery of natural products for the management of neurodegenerative diseases. J. Mol. Neurosci..

[45] Nishino S., Fujiki Y., Sato T., Kato Y., Shirai R., Oizumi H., Yamamoto M., Ohbuchi K., Miyamoto Y., Mizoguchi K. (2022). Hesperetin, a citrus flavonoid, ameliorates inflammatory cytokine-mediated inhibition of oligodendroglial cell morphological differentiation. Neurol. Int..

[46] Kato Y., Shirai R., Ohbuchi K., Oizumi H., Yamamoto M., Miyata W., Iguchi T., Mimaki Y., Miyamoto Y., Yamauchi J. (2023). Hesperetin ameliorates inhibition of neuronal and oligodendroglial cell differentiation phenotypes induced by knockdown of Rab2b, an autism spectrum disorder-associated gene product. Neurol. Int..

[47] Li D., Mitsuhashi S., Ubukata M. (2012). Protective effects of hesperidin derivatives and their stereoisomers against advanced glycation end-products formation. Pharm. Biol..

[48] Yu H., Haskins J.S., Su C., Allum A., Haskins A.H., Salinas V.A., Sunada S., Inoue T., Aizawa Y., Uesaka M. (2016). In vitro screening of radioprotective properties in the novel glucosylated flavonoids. Int. J. Mol. Med..

[49] Liang X., Draghi N.A., Resh M.D. (2004). Signaling from integrins to Fyn to Rho family GTPases regulates morphologic differentiation of oligodendrocytes. J. Neurosci..

[50] Madrigal M.P., Ballester-Lurbe B., Gómez O., Moreno-Bravo J.A., Puelles E., Jurado S., Garcia-Verdugo J.M., Pérez-Roger I., Terrado J. (2022). Rnd3 is necessary for the correct oligodendrocyte differentiation and myelination in the central nervous system. Brain Struct. Funct..

[51] Heng J.I., Nguyen L., Castro D.S., Zimmer C., Wildner H., Armant O., Skowronska-Krawczyk D., Bedogni F., Matter J.M., Hevner R. (2008). Neurogenin 2 controls cortical neuron migration through regulation of Rnd2. Nature.

[52] Armentano M., Filosa A., Andolfi G., Studer M. (2006). COUP-TFI is required for the formation of commissural projections in the forebrain by regulating axonal growth. Development.

[53] Demuro S., Di Martino R.M.C., Ortega J.A., Cavalli A. (2021). GSK-3b, FYN, and DYRK1A: Master regulators in neurodegenerative pathways. Int. J. Mol. Sci..

[54] Peng S., Fu Y. (2023). FYN: Emerging biological roles and potential therapeutic targets in cancer. J. Transl. Med..

[55] Ali M.Y., Jannat S., Jung H.A., Choi J.S. (2021). Structural bases for hesperetin derivatives: Inhibition of protein tyrosine phosphatase 1B, kinetics mechanism and molecular molecules. Molecules.

[56] Figlia G., Gerber D., Suter U. (2018). Myelination and mTOR. Glia.

[57] Ogata T., Iijima S., Hoshikawa S., Miura T., Yamamoto S., Oda H., Nakamura K., Tanaka S. (2004). Opposing extracellular signal-regulated kinase and Akt pathways control Schwann cell myelination. J. Neurosci..

[58] Dahl K.D., Almeida A.R., Hathaway H.A., Bourne J., Brown T.L., Finseth L.T., Wood T.L., Macklin W.B. (2023). mTORC2 loss in oligodendrocyte progenitor cells results in regional hypomyelination in the central nervous system. J. Neurosci..

[59] Lu Q., Lai Y., Zhang H., Ren K., Liu W., An Y., Yao J., Fan H. (2022). Hesperetin inhibits TGF-β1-induced migration and invasion of triple negative breast cancer MDA-MB-231 cells via suppressing Fyn/Paxillin/RhoA pathway. Integr. Cancer Ther..

[60] Lu Q., Kishi H., Zhang Y., Morita T., Kobayashi S. (2022). Hesperetin inhibits sphingosylphosphorylcholine-induced vascular smooth muscle contraction by regulating the Fyn/Rho-Kinase pathway. J. Cardiovasc. Pharmacol..

[61] Pepper R.E., Pitman K.A., Cullen C.L., Young K.M. (2018). How do cells of the oligodendrocyte lineage affect neuronal circuits to influence motor function, memory and mood?. Front. Cell. Neurosci..

[62] Sen M.K., Mahns D.A., Coorssen J.R., Shortland P.J. (2019). Behavioural phenotypes in the cuprizone model of central nervous system demyelination. Neurosci. Biobehav. Rev..

[63] Wegener A.J., Neigh G.N. (2021). Animal models of anxiety and depression: Incorporating the underlying mechanisms of sex differences in macroglia biology. Front. Behav. Neurosci..

[64] Xiong Y., Hong H., Liu C., Zhang Y.Q. (2023). Social isolation and the brain: Effects and mechanisms. Mol. Psychiatry.

