# Supplementary material for: Investigating the Protective Effects of a Citrus Flavonoid on the Retardation Morphogenesis of the Oligodendroglia-like Cell Line by Rnd2 Knockdown
Source: Neurol Int. 2023 Dec 26;16(1):33–61. doi: 10.3390/neurolint16010003 (PMC10801557; doi:10.3390/neurolint16010003)
Supplement: Supplementary file 1 [file neurolint-16-00003-s001.zip › Supplementary Figures and Table Legends.pdf]

Supplemental figure and table legends

A

gRNA sequences
gRnd2 113 sense: 5' GATCCGCACCCGTGCAAAAATGCAGGGGTCTAAAACCCACGGTGTTT-GAGAACTACACTTTTTTAT 3'
gRnd2 113 antisense: 5' CGATAAAAAAGTGTAGTTCTCAAACACCGTGGGTTTTAGACCCCTGCA-TTTTTGCACGGGTGCG 3'
gRnd2 250 sense: 5' GATCCGCACCCGTGCAAAAATGCAGGGGTCTAAAACATCTGCTTTGA-CATTAGCCGGCTTTTTTAT 3'
gRnd2 250 antisense: 5' CGATAAAAAAGCCGGCTAATGTCAAAGCAGATGTTTTAGACCCCTGCA-TTTTTGCACGGGTGCG 3'
**PCR primers**
Rnd2 (sense, Tm values = 55°C) 5' ATGTGGGATACTTCAGGTTCC 3'
Rnd2 (antisense, Tm values = 56°C) 5' TCACATGAGGTTACAGCTCTTG 3'
Actin (sense, Tm values = 66°C) 5' ATGGATGACGATATCGCTGCGCTGGTC 3'
Actin (antisense, Tm values = 66°C) 5' CTAGAAGCACTTGCGGTGCACGATGGAG 3'

B

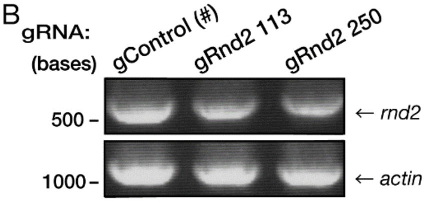

#Kato *et. al.* Non-Coding RNA (2022) Vol. 8, No. 3, page 42

Figure S1. Effects of knockdown of Rnd2 using the CRISPR/CasRx system. Knockdown efficiencies in transfected cells were determined by RT-PCR and agarose gel electrophoresis. The gRNA with nucleotide sequence starting at position 250 of Rnd2 was used in subsequent experiments.

A

siRNA sequences
siRnd2 156: 5' GCGCCGCAUUGAGCUCAAC-dTdT 3'<br>siRnd2 300: 5' GUGGCAAGGAGAGACUCAG-dTdT 3'
**PCR primers**
Rnd2 (sense, Tm values = 55°C) 5' ATGTGGGATACTTCAGGTTCC 3'<br>Rnd2 (antisense, Tm values = 56°C) 5' TCACATGAGGTTACAGCTCTTG 3'<br>Actin (sense, Tm values = 66°C) 5' ATGGATGACGATATCGCTGCGCTGGTC 3'<br>Actin (antisense, Tm values = 66°C) 5' CTAGAAGCACTTGCGGTGCACGATGGAG 3'

B

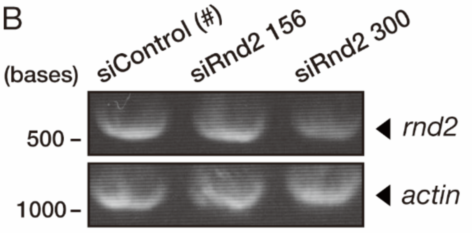

#Yamauchi *et. al.* Exp. Cell Res. (2009) Vol. 315, No. 12, pages 2043-2052

Figure S2. Effects of knockdown of Rnd2 using the RNA interference technique. Knockdown efficiencies in transfected cells were determined by RT-PCR and agarose gel electrophoresis. The siRNA with sequence starting at position 300 of Rnd2 was used in subsequent experiments.

A

siRNA sequences
siPRAG1 105: 5' GGCUAGAGCCAACAGCCUA-dTdT 3'<br>siPRAG1 178: 5' GGCGUGAAUGGCUUGGCCU-dTdT 3'<br>siPRAG1 204: 5' GCCCACCAUUGCUGUAAAG-dTdT 3'
**PCR primers**
PRAG1 (sense, Tm values = 62°C) 5' ATGTCTGCGTGCAGCGACTTTG 3'<br>PRAG1 (antisense, Tm values = 62°C) 5' CGGTTCCTTCACAAGAGAGCAGTAGTC 3'<br>Actin (sense, Tm values = 66°C) 5' ATGGATGACGATATCGCTGCGCTGGTC 3'<br>Actin (antisense, Tm values = 66°C) 5' CTAGAAGCACTTGCGGTGCACGATGGAG 3'

B

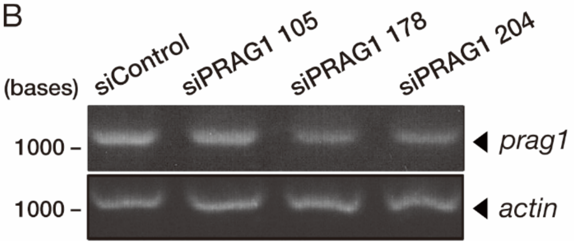

Figure S3. Effects of knockdown of Prag1 using the RNA interference technique. Knockdown efficiencies in transfected cells were determined by RT-PCR and agarose gel electrophoresis. The siRNA with sequence starting at position 178 of Prag1 was used in subsequent experiments.

A

PCR primers
Fyn (sense, Tm values = 61°C) 5' ATGGGCTGTGTGCAATGTAAGGATAAAG 3'
Fyn (antisense, Tm values = 60°C) 5' GGTTTGGCAACTTCAGAGCTCTTC 3'
Actin (sense, Tm values = 66°C) 5' ATGGATGACGATATCGCTGCGCTGGTC 3'
Actin (antisense, Tm values = 66°C) 5' CTAGAAGCACTTGCGGTGCACGATGGAG 3'

B

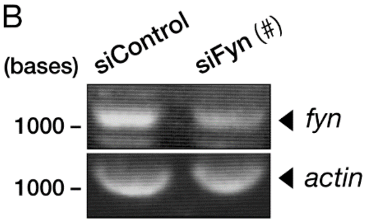

#sc-35425 (Mouse Fyn siRNA, Santa Cruz Biotechnology, Santa Cruz, CA, USA)

Figure S4. Effects of knockdown of Fyn using the RNA interference technique. Knockdown efficiencies in transfected cells were determined by RT-PCR and agarose gel electrophoresis. The available siRNA for Fyn was used in subsequent experiments.

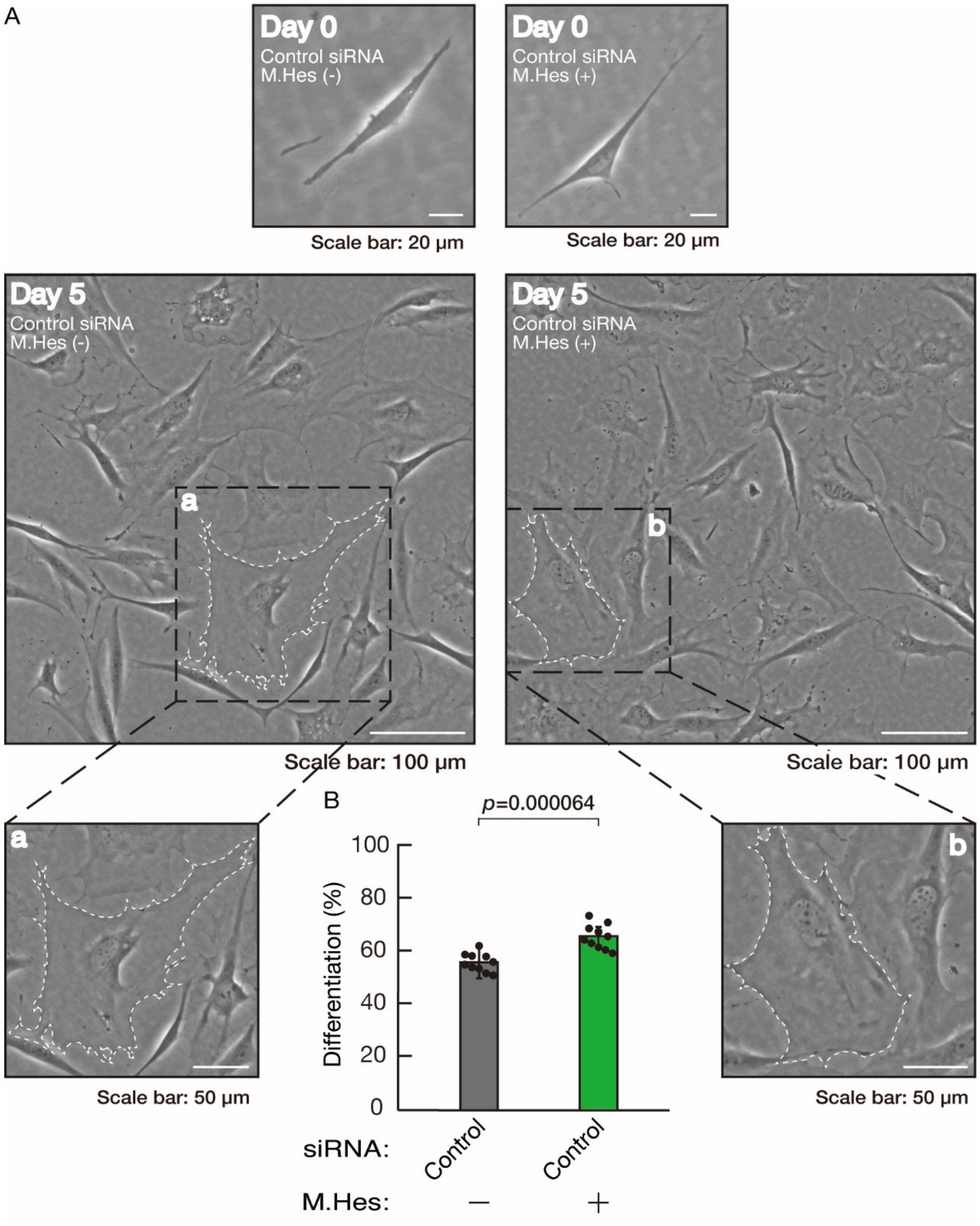

Figure S5. Effects of monoglucosyl hesperidine on morphological differentiation in control knockdown conditions. (A, B) Cells were transfected with siRNA for control (luciferase) in the presence or absence (vehicle control) of monoglucosyl hesperidine (M.Hes). Following the induction of differentiation, differentiated cells in the M.Hes(+)

and M.Hes(-) groups were counted and analyzed statistically (** $p<0.01$; n = 10 fields). The typical cells with or without widespread membranes were surrounded with dashed lines. The inset images (a, b) are magnified from the squares in the large images.

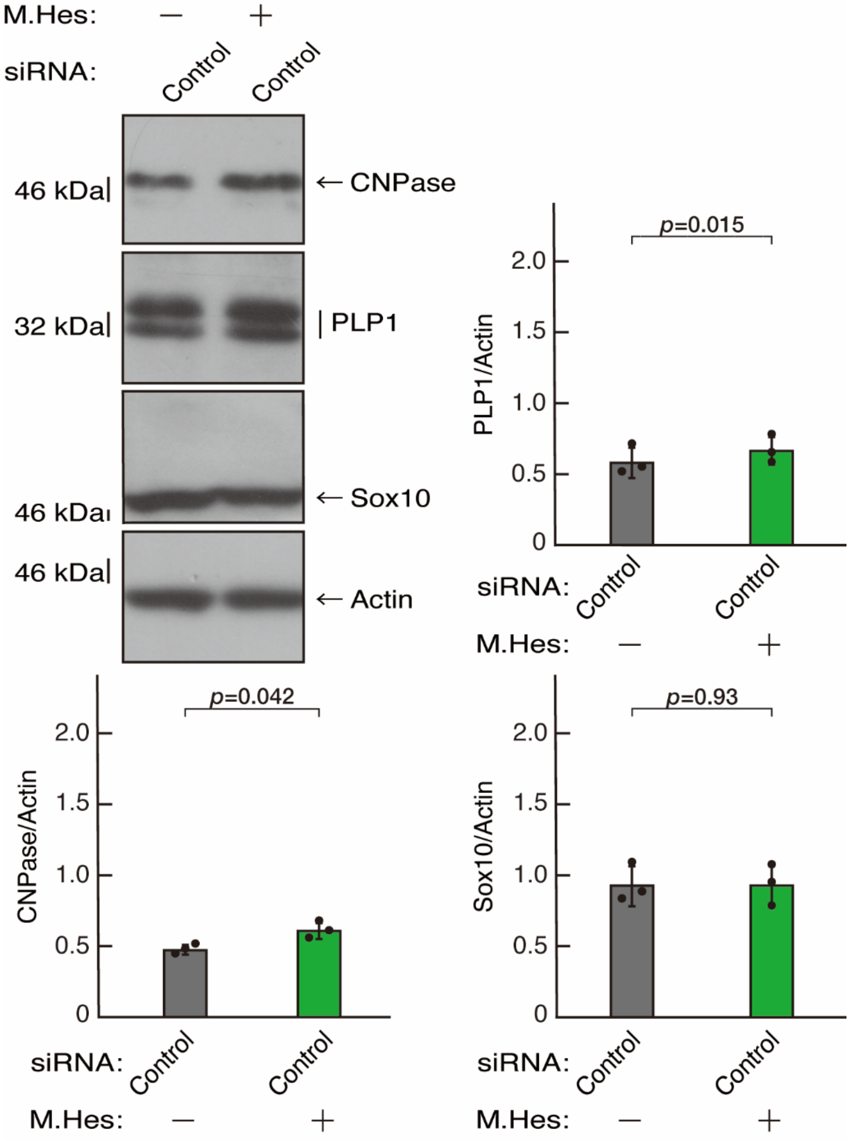

Figure S6. Effects of monoglucosyl hesperidine on expression levels of differentiation markers in control knockdown conditions. Cells were transfected with siRNA for control (luciferase) in the presence or absence (vehicle control) of monoglucosyl hesperidine

(M.Hes). Following the induction of differentiation, immunoblots for CNPase, PLP1, Sox10, and actin at 5 days were analyzed and their expression levels were statistically depicted as immunoreactive bands normalized to actin ones. (n = 3 blots).

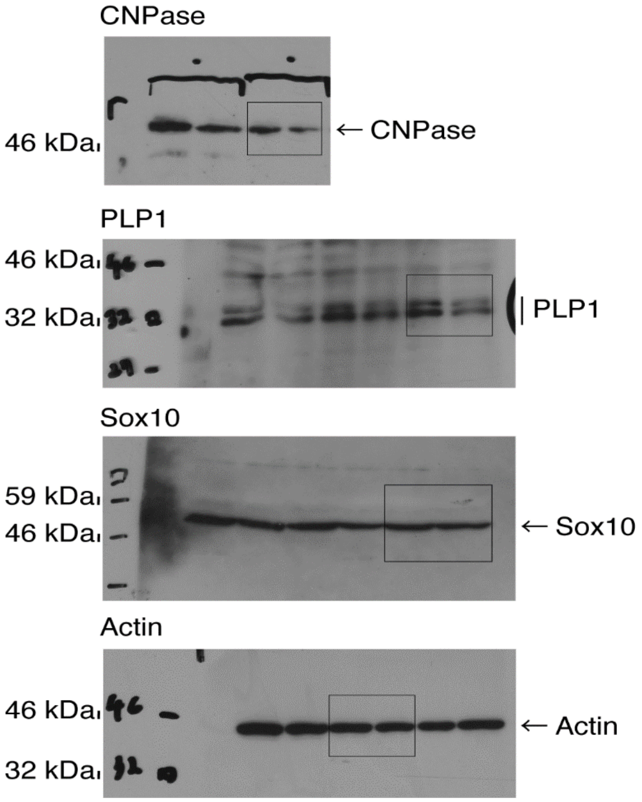

Figure S7. Original size gels saved as TIFF files of Figure 2.

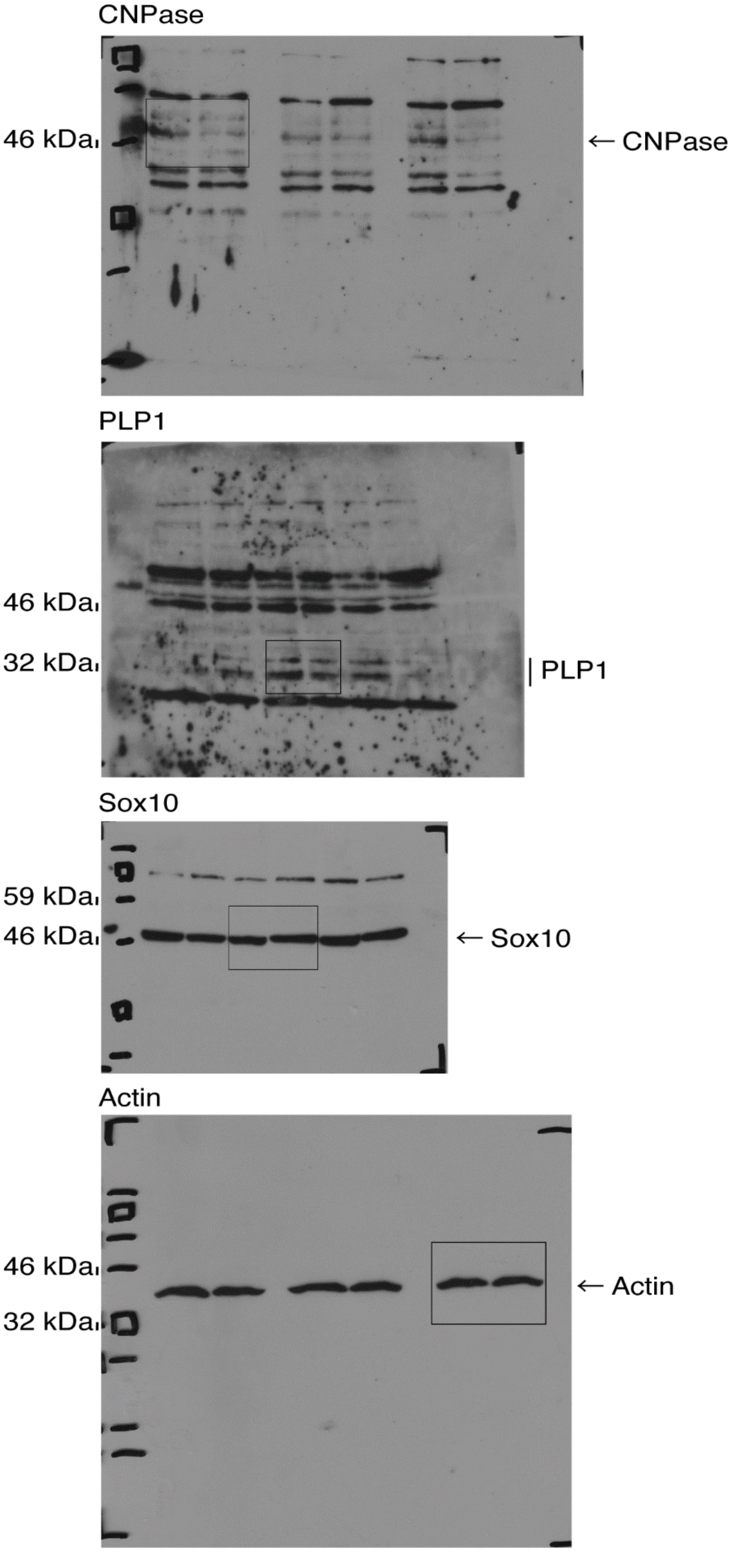

Figure S8. Original size gels saved as TIFF files of Figure 4.

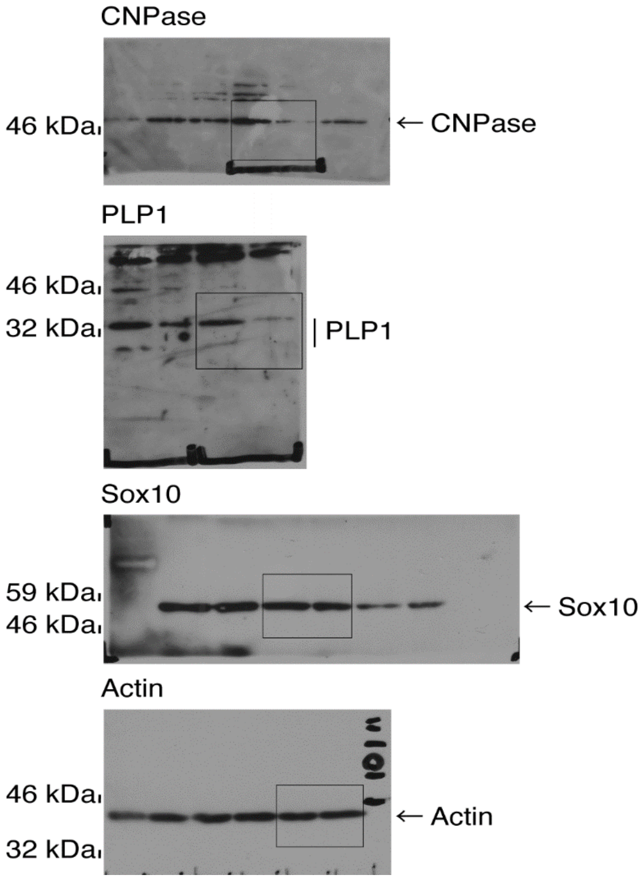

Figure S9. Original size gels saved as TIFF files of Figure 6.

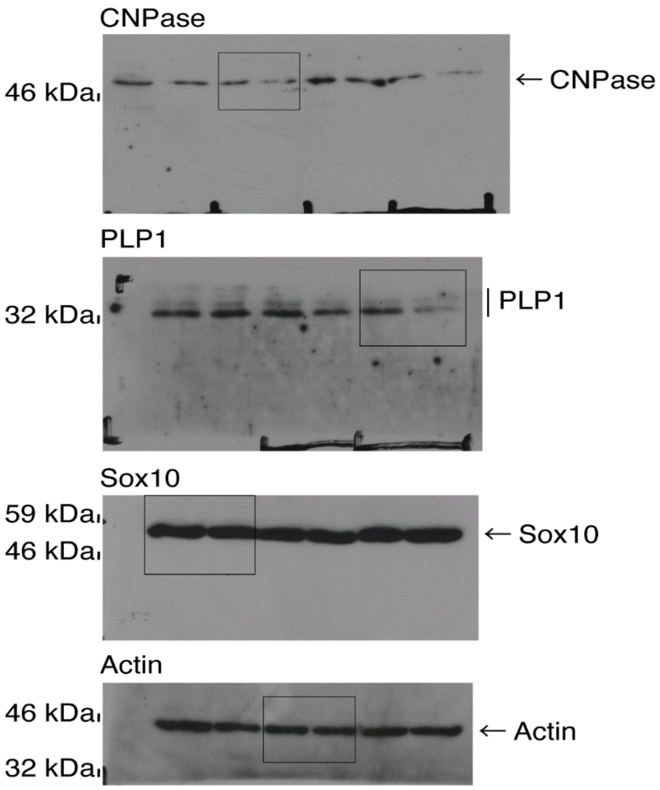

Figure S10. Original size gels saved as TIFF files of Figure 8.

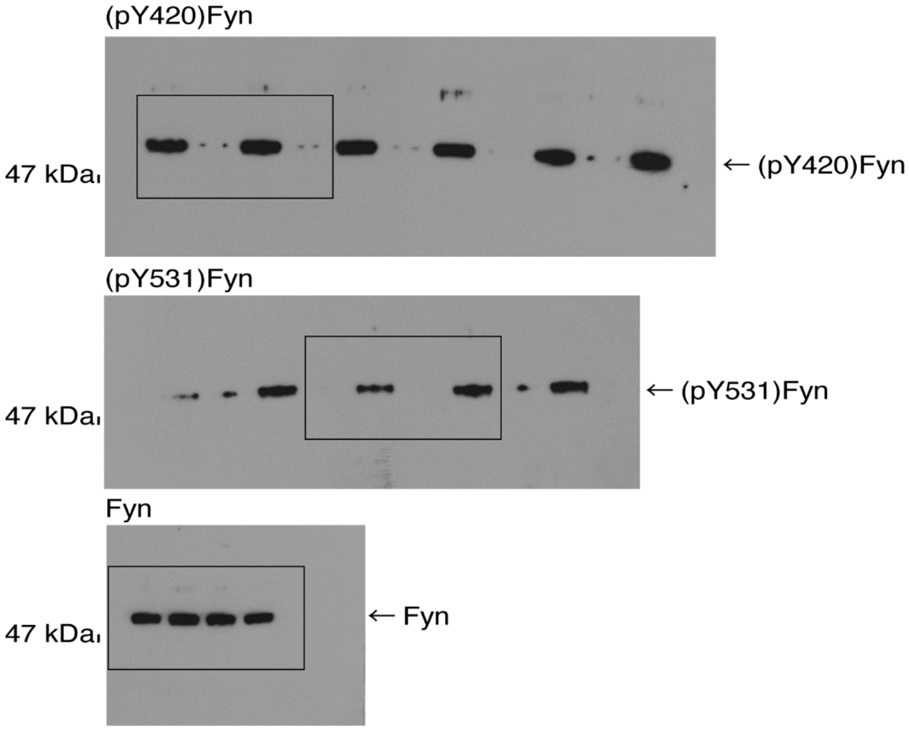

Figure S11. Original size gels saved as TIFF files of Figure 9.

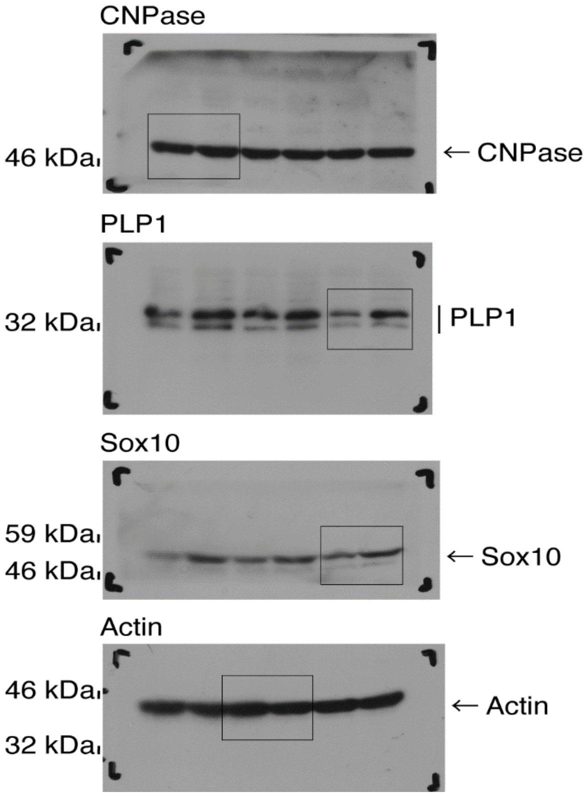

Figure S12. Original size gels saved as TIFF files of Figure 11.

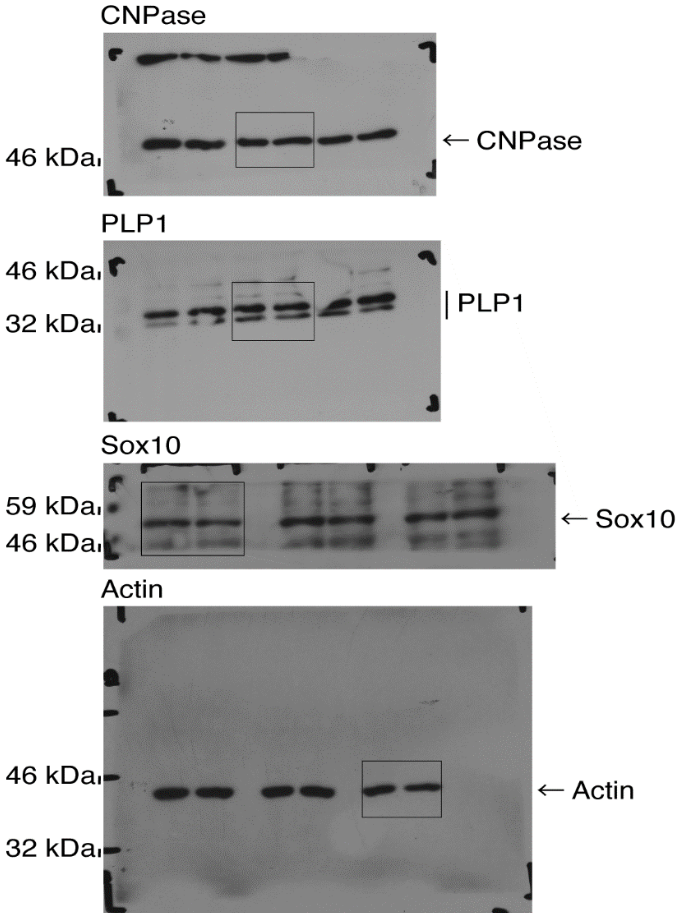

Figure S13. Original size gels saved as TIFF files of Figure 13.

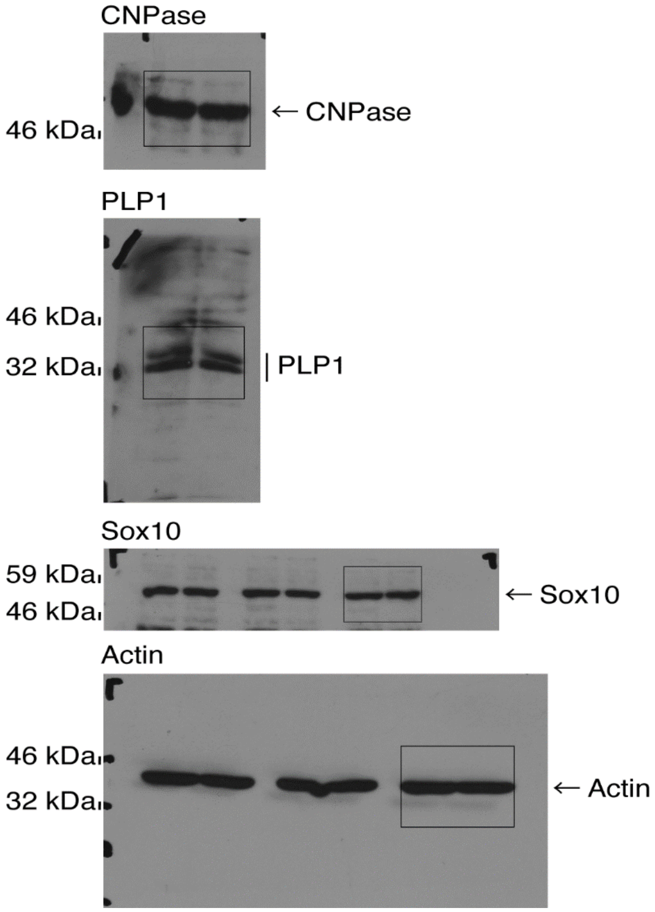

Figure S14. Original size gels saved as TIFF files of Figure 15.

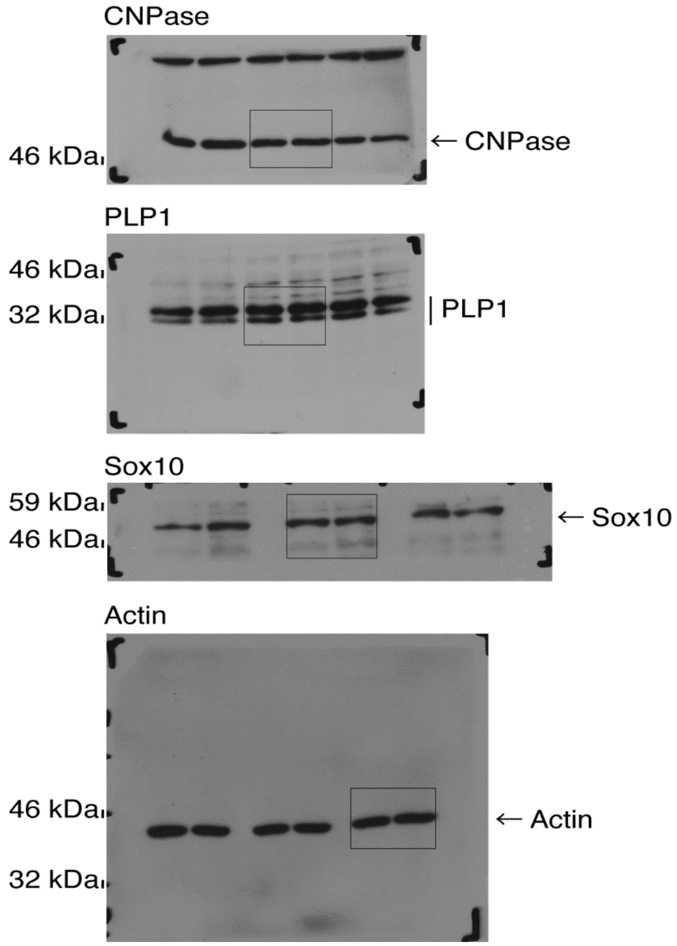

Figure S15. Original size gels saved as TIFF files of Figure 17.

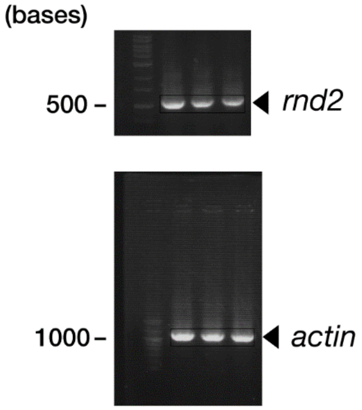

Figure S16. Original size gels of Figure S1.

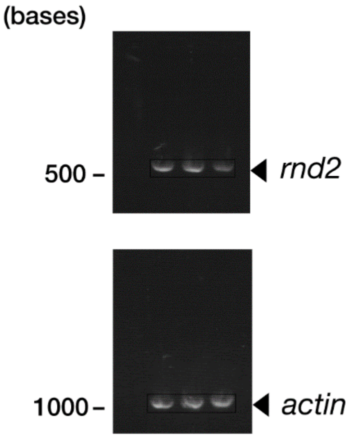

Figure S17. Original size gels of Figure S2.

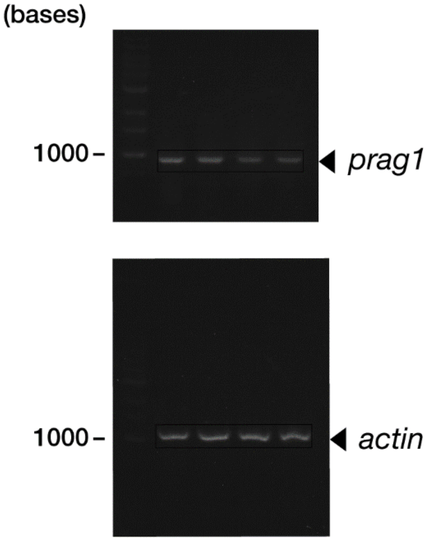

Figure S18. Original size gels of Figure S3.

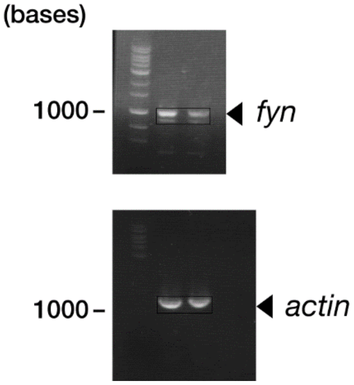

Figure S19. Original size gels of Figure S4.

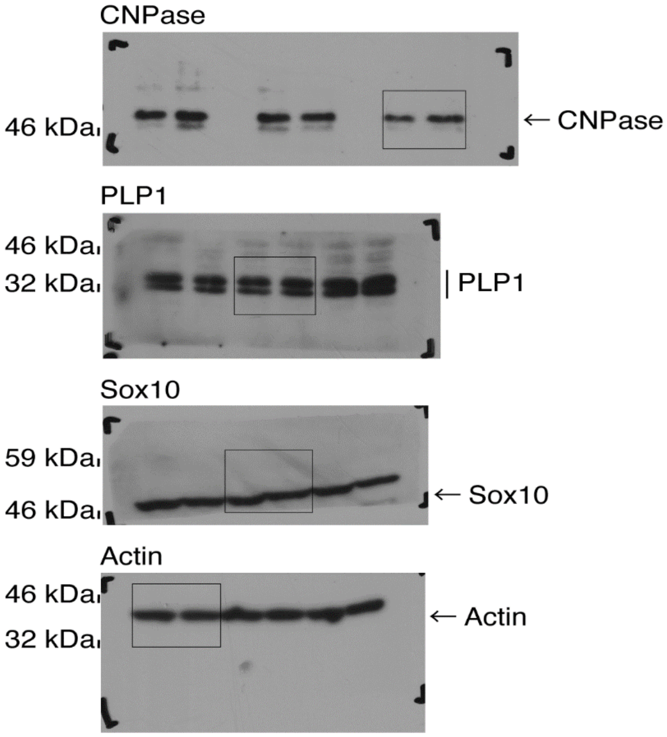

Figure S20. Original size gels of Figure S6.

Table S1. Statistical data for Figure 1.

Table S2. Statistical data for Figure 2.

Table S3. Statistical data for Figure 3.

Table S4. Statistical data for Figure 4.

Table S5. Statistical data for Figure 5.

Table S6. Statistical data for Figure 6.

Table S7. Statistical data for Figure 7.

Table S8. Statistical data for Figure 8.

Table S9. Statistical data for Figure 10.

Table S10. Statistical data for Figure 11.

Table S11. Statistical data for Figure 12.

Table S12. Statistical data for Figure 13.

Table S13. Statistical data for Figure 14.

Table S14. Statistical data for Figure 15.

Table S15. Statistical data for Figure 16.

Table S16. Statistical data for Figure 17.

Table S17. Statistical data for Figure S5.

Table S18. Statistical data for Figure S6.
